# Direct Phasing of Protein Crystals with Hybrid Difference Map Algorithms

**DOI:** 10.3390/molecules31030472

**Published:** 2026-01-29

**Authors:** Hongxing He, Yang Liu, Wu-Pei Su

**Affiliations:** 1 Department of Physics, School of Physical Science and Technology, Ningbo University, Ningbo 315211, China; 2311690029@nbu.edu.cn; 2 Department of Physics and Texas Center for Superconductivity, University of Houston, Houston, TX 77204, USA

**Keywords:** direct methods, phase retrieval, hybrid difference map, protein crystallography, iterative projection algorithm

## Abstract

Direct methods for solving protein crystal structures from X-ray diffraction data provide an essential approach for validating predicted models while avoiding external model bias. Nevertheless, traditional iterative projection algorithms, including the widely used Difference Map (DiffMap), are often limited by modest phase retrieval success rates. To address this limitation, we introduce a novel Hybrid Difference Map (HDM) algorithm that synergistically combines the strengths of DiffMap and the Hybrid Input–Output (HIO) method through six distinct iterative update rules. HDM retains an optimized DiffMap-style relaxation term for fine-grained density modulation in protein regions while adopting HIO’s efficient negative feedback mechanism for enforcing the solvent flatness constraint. Using the transmembrane photosynthetic reaction center 2uxj as a test case, the first HDM formula, HDM-f1, successfully recovered an atomic-resolution structure directly from random phases under a conventional full-resolution phasing scheme, demonstrating the robust phasing capability of the approach. Systematic evaluation across 22 protein crystal structures (resolution 1.5–3.0 Å, solvent content ≥ 60%) revealed that all six HDM variants outperformed DiffMap, achieving 1.8–3.5× higher success rates (average 2.8×), performing on par with or exceeding HIO under a conventional phasing scheme. Further performance gains were achieved by integrating HDM with advanced strategies: resolution weighting and a genetic algorithm-based evolutionary scheme. The genetic evolution strategy boosted the success rate to nearly 100%, halved the median number of iterations required for convergence, and reduced the final phase error to approximately 35° on average across test structures through averaging of multiple solutions. The resulting electron density maps were of high interpretability, enabling automated model building that produced structures with a backbone RMSD of less than 0.5 Å when compared to their PDB-deposited counterparts. Collectively, the HDM algorithm suite offers a robust, efficient, and adaptable framework for direct phasing, particularly for challenging cases where conventional methods struggle. Our implementation supports all space groups providing an accessible tool for the broader structural biology community.

## 1. Introduction

X-ray crystallography remains one of the primary experimental techniques for determining three-dimensional macromolecular structures. However, crystallographic experiments record only diffraction intensities while the crucial phase information is lost, giving rise to the well-known phase problem. Reconstruction of real-space electron density maps requires both amplitude and phase information. Traditional experimental phasing methods, such as isomorphous replacement (SIR/MIR) or anomalous scattering (SAD/MAD), rely on heavy-atom derivatization or selenomethionine substitution, processes that are experimentally demanding and may yield inadequate derivative data for certain proteins. Molecular replacement utilizes homologous structures or AlphaFold-predicted models to provide initial phases but may introduce model bias [1,2,3]. In contrast, direct methods start from random phases and utilize only the protein’s native crystallographic diffraction amplitudes, progressively recovering the lost phase information through iterative projection algorithms. The resulting models are free from external model bias, making direct methods a powerful tool for *ab initio* structure determination of novel proteins and for validating the accuracy of predicted structures.

The recent revolution in AI-based structure prediction, exemplified by AlphaFold [1] and related tools [2,3], has transformed structural biology by providing accurate predicted models for molecular replacement. However, these AI-derived phases inherit the inherent biases of the prediction models, potentially propagating errors into the final structures. Direct phasing methods offer a complementary, model-independent approach: they derive phases solely from experimental diffraction data without relying on external structural templates. This independence makes direct methods uniquely valuable in several contexts: validating AI-predicted models against experimental data, identifying local structural errors or conformational differences between predicted and true structures, and providing unbiased experimental phases when predictions may be unreliable—for example, in novel folds, intrinsically disordered regions, or ligand-induced conformational changes. Thus, rather than competing with AI-assisted pipelines, direct phasing methods serve as an essential validation and quality-control tool in the modern structural biology workflow.

The application of direct methods to protein crystallography has a rich history. The concept originated from small-molecule crystallography [4,5,6,7,8,9,10] and was later adapted for macromolecules. Seminal algorithms such as the Hybrid Input–Output (HIO) method proposed by Fienup [11] and the Difference Map (DiffMap) algorithm introduced by Elser [12,13] established foundational frameworks for iterative phase retrieval. Early applications to crystallographic phase problems were explored by Millane and colleagues, who applied dual-space iterative projections with the HIO algorithm for phase retrieval in crystallography [14,15,16], while Miao et al. demonstrated phase retrieval for non-periodic structures [17]. Lunin et al. exploited electron density connectivity to *ab initio* solve low-resolution protein crystal structures [18], and Marchesini provided a comprehensive analysis of various iterative projection algorithms for phase recovery [19]. Su retrieved low- and medium-resolution structural features of macromolecules directly from the diffraction intensities [20].

Subsequent developments integrated additional constraints to enhance convergence for macromolecular crystals. Liu et al. combined small-angle X-ray scattering (SAXS) molecular envelopes with HIO, histogram matching, and non-crystallographic symmetry (NCS) averaging to successfully solve protein structures [21]. He and Su employed weighted density averaging to automatically reconstruct protein envelopes during *ab initio* HIO-based phase retrieval in protein crystallography [22], while Lo and Millane applied the difference map algorithm to direct phasing of protein and virus crystals [23,24,25]. He et al. further expanded the scope by developing algorithms for *ab initio* NCS density averaging [26], and introduced a transition region between protein and solvent domains to refine envelope reconstruction [27]. Kingston et al. established a more automated pipeline utilizing the DiffMap algorithm in direct phasing of protein crystal structures [28,29]. Building upon these advances, we previously incorporated genetic algorithms into the direct phasing process [30], enabling structure solution across broader ranges of solvent content and resolution. In parallel, Pan et al. demonstrated a machine learning-based approach to protein structure determination from diffraction data with partial template information [31].

Among the various iterative algorithms, HIO and DiffMap are the most commonly used for density modification. The HIO algorithm excels in the solvent region by introducing a negative feedback mechanism, which efficiently exploits the constraint of approximately uniform electron density to drive the iteration and prevent stagnation in local minima. However, its update rule in the protein region is identical to the simple Error Reduction algorithm, lacking sophisticated density modulation. Conversely, the DiffMap algorithm employs a relaxation term derived from the difference between two candidate solutions, acting over the entire unit cell and enhancing the global search capability to escape local minima. Nevertheless, its update strategy in the protein region does not fully exploit dual-space constraints, and its relaxation term in the solvent region is less efficient than HIO’s tunable negative feedback. Therefore, the primary motivation for this study is to synergistically combine the strengths of HIO and DiffMap while circumventing their weaknesses, ultimately developing a novel, more effective iterative algorithm to enrich the toolbox for *ab initio* phasing.

Existing iterative methods based on HIO and DiffMap typically require crystals with high solvent content and high diffraction resolution [22,28,32]. As the solvent content decreases, the constraint provided by the constant solvent density weakens, making phase recovery progressively more difficult. Lower resolution blurs the boundary between the protein and solvent regions, causing the density at the solvent edge to deviate from a constant, further complicating phasing. Additionally, missing low-angle diffraction data or significant measurement errors pose extra challenges for reconstructing an accurate molecular envelope. In summary, the success rate of algorithms like HIO and DiffMap is influenced by a combination of factors including solvent content, resolution, data completeness, data quality, and the complexity of the protein envelope within the unit cell. Developing hybrid algorithms that leverage the respective advantages of HIO and DiffMap to provide a richer set of iterative options is therefore of significant theoretical importance and practical necessity for solving novel structures.

To address this need, we present a new family of Hybrid Difference Map (HDM) algorithms, comprising six distinct iterative formulas. The core innovation of HDM lies in its partitioned update strategy: it retains and optimizes the relaxation term of DiffMap for fine density modulation in the protein region, while incorporating the efficient, tunable negative feedback mechanism of HIO in the solvent region. This hybrid approach enables more effective density control, particularly beneficial for crystals with limited solvent content. Systematic tests on 22 real protein diffraction datasets, spanning various space groups, demonstrate that the HDM series algorithms achieve a success rate more than double that of traditional DiffMap, reaching or even surpassing the performance of HIO. Furthermore, when combined with our previously developed phasing strategies—the conventional full-resolution strategy, the resolution-weighted strategy [33], and the genetic evolution strategy [30]—the success rate of HDM can be boosted to nearly 100%, with the median number of iterations required for convergence reduced by more than half. The enhanced success rate also allows for averaging multiple independently converged electron density maps, further reducing the final phase error to approximately $35^{{}^{\circ}}$ and yielding high-quality maps directly suitable for automated model building, producing atomic models with a backbone RMSD of less than 0.5 Å against the PDB-deposited structures. However, it is important to note that, like other direct phasing algorithms, HDM performance remains dependent on crystal solvent content, typically requiring values around or above 60% for optimal results.

This paper is structured as follows. We will first detail the design and derivation of the six novel updating formulas constituting the HDM algorithm series. Subsequently, we will present a complete case study of *ab initio* structure determination using HDM-f1 to illustrate its practical phasing capability. Furthermore, we will demonstrate the robust performance of all HDM formulas across different phasing strategies through comprehensive tests and comparisons with HIO and DiffMap. Finally, we will discuss the advantages, limitations, and future potential of the HDM approach, highlighting its contribution as a new, efficient, and versatile tool for direct phasing in protein crystallography.

## 2. Materials and Methods

### 2.1. Dataset Selection and Preprocessing

To ensure broad and statistically robust evaluation of the algorithms, we constructed a test set comprising 22 protein crystal structures sourced from the Protein Data Bank (PDB), with detailed descriptions provided in Appendix A, Table A1. All structures exhibit diffraction resolutions between 1.5 Å and 3.0 Å, spanning from high-resolution regimes that reveal atomic details to the more challenging medium-resolution range. Furthermore, the estimated solvent content based on the Matthews coefficient is generally no less than 60%, representing the empirical lower bound for conventional iterative projection algorithms and allowing a rigorous assessment of the proposed method under critical conditions. To guarantee data reliability, the reported $R_{work}$ of the selected datasets is typically below 0.22 (with the exception of 9ee7, $R_{work}=0.298$). Structural diversity was also carefully considered: the set includes multiple space groups, such as C121, H32, I121, I4_1_22, P2_1_2_1_2_1_, P3_1_21, P321, P3_2_21, P4_1_2_1_2, P4_1_22, P4_1_32, P4_3_2_1_2 and P6_1_22, covering crystal systems from monoclinic, orthorhombic, and trigonal to tetragonal, hexagonal, and cubic. In addition, the molecular sizes vary considerably, with the number of residues per asymmetric unit ranging from 88 to 825, corresponding to non-hydrogen atom counts between 690 and 7707. This diversity ensures a comprehensive examination of the algorithm’s adaptability to varying crystal symmetries and structural complexities.

**Figure 1:**
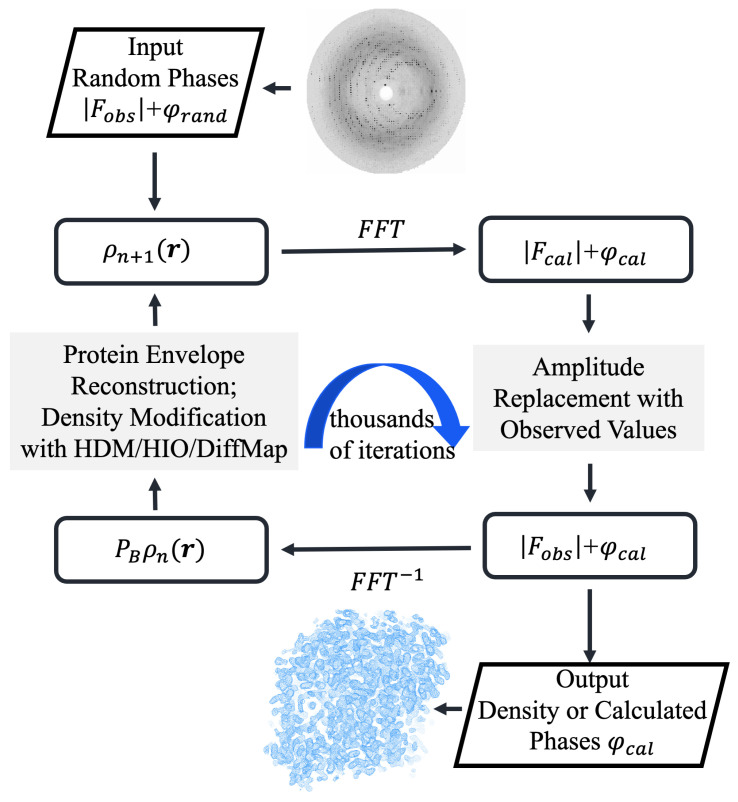
Flowchart of direct phasing with HDM/HIO/DiffMap algorithms. The process initiates from random phases or random electron density, iterating between real space (reconstructing the protein envelope and applying HDM/HIO/DiffMap density modification) and reciprocal space (replacing calculated amplitudes with observed diffraction amplitudes). After thousands of iterations, the converged electron density or calculated phases are output.

The data preprocessing workflow was carried out using well-established software tools including CCP4 (v9.0) [34], and Phenix (v1.16-3549) [35] to maintain standardization and reproducibility. Diffraction amplitude files in .cif format, as obtained from the PDB, were first converted into MTZ-format files (sf.mtz) using the cif2mtz utility from the CCP4 suite (v9) [34]. That file served as the primary input for all subsequent phase retrieval steps. For real-space histogram matching [36], a reference electron density distribution was generated for each target structure. To implement the histogram matching constraint in real space, an optional reference structure, such as a known homologous structure or an AI-predicted model, can be provided. When available, Phenix.fmodel [37] is used to calculate structure factors from that reference, incorporating bulk solvent correction and adjusting the temperature factor according to the target structure’s resolution to generate a histogram reference file. For each test structure in this study, we used its own PDB coordinate file as input to compute the reference histogram with bulk solvent correction. It is important to emphasize that in a true *ab initio* phasing scenario, the algorithm operates independently of atomic coordinates, requiring only the diffraction data in MTZ format. As demonstrated by Zhang and Main [36], electron density histograms at a given resolution are remarkably similar across different protein structures; therefore, a histogram derived from any homologous structure, AI-predicted model, or even an unrelated protein of similar molecular weight can serve as an effective reference for constraining protein region density. The reference histogram is generated only once prior to phase retrieval, and during iteration, the matching is performed at a coarse-grained level, adjusting the overall density distribution rather than imposing atom-specific constraints. For benchmarking purposes in this study, the use of target PDB coordinates for histogram generation does not affect our conclusions, as these histograms merely provide statistically representative protein density distributions rather than structural information. Figure 1 demonstrates the flowchart of the algorithm. The code was developed using the Clipper C++ libraries for crystallographic computing [38].

To monitor potential overfitting throughout the iterative process, 1% of the diffraction data were randomly assigned as a free set. These reflections were excluded from all real-space and reciprocal-space constraint enforcement and were used exclusively for computing $R_{\mathrm{free}}$. Finally, for quantitative assessment of the retrieved phases, the true structure factor phases derived from the PDB coordinates with bulk solvent contribution were stored in an fmodel.mtz file, enabling calculation of the mean phase error (Δφ).

### 2.2. HDM Hybrid Iterative Projection Algorithm Design

#### 2.2.1. Theoretical Basis: Motivation for Fusion from DiffMap and HIO to HDM

Phase retrieval in direct phasing is essentially a problem of finding the global optimum under non-convex constraint sets. Let the real-space constraint set be A (including flat density in bulk solvent and similar density distribution in protein region) and the Fourier-space constraint set be B (experimental diffraction amplitudes), with their projection operators denoted as $P_{A}$ and $P_{B}$, respectively.

The Difference Map (DiffMap) algorithm [12] enhances global search capability by constructing two complementary candidate solutions shown in Equation (1):

(1) $$\begin{array}{cc} \rho_{A}^{n} & =P_{A}\left[\left(1+\beta_{0}^{-1}\right)P_{B}\rho_{n}-\beta_{0}^{-1}\rho_{n}\right]\;\mathrm{and}\;\rho_{B}^{n}=P_{B}\left[\left(1-\beta_{0}^{-1}\right)P_{A}\rho_{n}+\beta_{0}^{-1}\rho_{n}\right] \end{array}$$

Its iterative update rule is shown in Equation (2):

(2) $$\begin{array}{cc} \rho_{n+1} & =\rho_{n}+\beta_{0}\left(\rho_{A}^{n}-\rho_{B}^{n}\right) \end{array}$$

The relaxation parameter $\beta_{0}$ is typically chosen from the interval (0.5, 1.0), controlling the step size. The relaxation term $\left(\rho_{A}^{n}-\rho_{B}^{n}\right)$ in DiffMap acts on the entire asymmetric unit, helping to escape local minima. However, as we will demonstrate later, its update in the protein region does not explicitly utilize the synergy of dual-space projections, and its feedback efficiency in the solvent region needs improvement. Specifically, when $\beta_{0}=1$, the DiffMap update in the solvent region reduces to $\rho_{n+1}=\rho_{n}-P_{B}\rho_{n}$, which corresponds to a fixed negative feedback factor of unity—larger than the optimal range of 0.7–0.9 typically used for γ in the Hybrid Input–Output method, resulting in overly aggressive corrections that can destabilize convergence.

The Hybrid Input–Output (HIO) algorithm [11] employs a partitioning strategy, as defined in Equation (3):

(3) $$\begin{array}{cc} \rho_{n+1}\left(r\right) & =\left\{\begin{array}{cc} P_{A}P_{B}\rho_{n}\left(r\right), & r\in S\\ \rho_{n}\left(r\right)-\gamma P_{B}\rho_{n}\left(r\right), & r\notin S \end{array}\right. \end{array}$$

Here, *S* denotes the protein region within the crystal asymmetric unit, and the negative feedback parameter γ is typically chosen from the interval (0.5, 1.0). Within the protein region *S*, we have incorporated the $P_{A}P_{B}$ projections into the HIO update formula. The negative feedback mechanism of HIO in the solvent region efficiently utilizes the ‘solvent flat’ constraint, driving the iteration towards the correct solution and effectively preventing stagnation. However, its treatment of the protein region is the same as the simple Error Reduction algorithm, lacking the dynamic modulation capability of DiffMap. The implementation of the HDM algorithm aims to inherit the relaxation advantage of DiffMap in the protein region while retaining the feedback efficiency of HIO in the solvent region, achieving partitioned and precise regulation.

#### 2.2.2. HDM Algorithm Derivation and Implementation Details

The implementation of HDM begins with the determination of the molecular envelope. At the $n^{th}$ iteration, we compute a Gaussian-weighted average of the current electron density $\rho_{n}$ to approximately locate the molecular boundary:

(4) $$w_{n}\left(r\right)=\int \rho_{n}\left(r^{\prime }\right)G(|r-r^{\prime }\left|;\sigma \right)\;dr^{\prime },$$

where G(r;σ) denotes a Gaussian kernel with standard deviation σ. The value of σ decreases linearly from 4.0 Å to 2.5 Å during iteration, enabling a coarse-to-fine strategy that captures the approximate envelope early and refines the boundary later. A threshold for $w_{n}$ is then set based on the solvent content estimated from the Matthews coefficient, classifying grid points into protein region (*S*) or solvent region accordingly.

Based on the above partitioning, starting from the special case of the DiffMap algorithm with $\beta_{0}=1$, and by partitioning and introducing the HIO concept while optimizing the update term in the protein region, we systematically derived six HDM iterative formulas. The core derivation path is as follows. Starting Point: When $\beta_{0}=1$, the DiffMap algorithm simplifies to Equation (5):

(5) $$\begin{array}{c} \rho_{n+1}\left(r\right)=\rho_{n}\left(r\right)+\left[\left(2P_{A}P_{B}-P_{A}-P_{B}\right)\rho_{n}\left(r\right)\right]\;(\mathrm{for}\;\mathrm{all}\;r) \end{array}$$

Considering that in the solvent region, the $P_{A}$ operator sets the density to zero, the partitioned form can be derived as Equation (6):

(6) $$\begin{array}{cc} \rho_{n+1}\left(r\right) & =\left\{\begin{array}{cc} \rho_{n}\left(r\right)+\left(2P_{A}P_{B}-P_{A}-P_{B}\right)\rho_{n}\left(r\right), & r\in S\\ \rho_{n}\left(r\right)-P_{B}\rho_{n}\left(r\right), & r\notin S \end{array}\right. \end{array}$$

Key Improvement 1 (HDM-f1, f2): In the protein region of Equation (6), we replace the first term $\rho_{n}\left(r\right)$ with the more constrained $P_{A}P_{B}\rho_{n}\left(r\right)$, yielding Equation (7):

(7) $$\begin{array}{cc} \rho_{n+1}\left(r\right) & =\left\{\begin{array}{cc} P_{A}P_{B}\rho_{n}\left(r\right)+\left(2P_{A}P_{B}-P_{A}-P_{B}\right)\rho_{n}\left(r\right), & r\in S\\ \rho_{n}\left(r\right)-P_{B}\rho_{n}\left(r\right), & r\notin S \end{array}\right.\;\left(\mathrm{HDM}\text{-}f1\right) \end{array}$$

Introducing tunable parameters β and γ gives Equation (8):

(8) $$\begin{array}{cc} \rho_{n+1}\left(r\right) & =\left\{\begin{array}{cc} P_{A}P_{B}\rho_{n}\left(r\right)+\beta \left(2P_{A}P_{B}-P_{A}-P_{B}\right)\rho_{n}\left(r\right), & r\in S\\ \rho_{n}\left(r\right)-\gamma P_{B}\rho_{n}\left(r\right), & r\notin S \end{array}\right.\;\left(\mathrm{HDM}\text{-}f2\right) \end{array}$$

When β=1,γ=1, the above equation reduces to HDM-f1 without explicit parameters.

Key Improvement 2 (HDM-f3, f4) and 3 (HDM-f5, f6): We note that in the protein region, $\left(\rho_{n}-P_{B}\rho_{n}\right)$ and $\left(P_{A}P_{B}\rho_{n}-P_{A}\rho_{n}\right)$ are small quantities near the solution. Neglecting $\left(\rho_{n}-P_{B}\rho_{n}\right)$ in Equation (6) yields Equation (9):

(9) $$\begin{array}{cc} \rho_{n+1}\left(r\right) & =\left\{\begin{array}{cc} P_{A}P_{B}\rho_{n}\left(r\right)+\left(P_{A}P_{B}-P_{A}\right)\rho_{n}\left(r\right), & r\in S\\ \rho_{n}\left(r\right)-P_{B}\rho_{n}\left(r\right), & r\notin S \end{array}\right.\;\left(\mathrm{HDM}\text{-}f3\right) \end{array}$$

Introducing tunable parameters β and γ gives Equation (10):

(10) $$\begin{array}{cc} \rho_{n+1}\left(r\right) & =\left\{\begin{array}{cc} P_{A}P_{B}\rho_{n}\left(r\right)+\beta \left(P_{A}P_{B}-P_{A}\right)\rho_{n}\left(r\right), & r\in S\\ \rho_{n}\left(r\right)-\gamma P_{B}\rho_{n}\left(r\right), & r\notin S \end{array}\right.\;\left(\mathrm{HDM}\text{-}f4\right) \end{array}$$

Neglecting $\left(P_{A}P_{B}\rho_{n}-P_{A}\rho_{n}\right)$ in Equation (7) yields Equation (11):

(11) $$\begin{array}{cc} \rho_{n+1}\left(r\right) & =\left\{\begin{array}{cc} P_{A}P_{B}\rho_{n}\left(r\right)+\left(P_{A}P_{B}-P_{B}\right)\rho_{n}\left(r\right), & r\in S\\ \rho_{n}\left(r\right)-P_{B}\rho_{n}\left(r\right), & r\notin S \end{array}\right.\;\left(\mathrm{HDM}\text{-}f5\right) \end{array}$$

Introducing tunable parameters β and γ gives Equation (12):

(12) $$\begin{array}{cc} \rho_{n+1}\left(r\right) & =\left\{\begin{array}{cc} P_{A}P_{B}\rho_{n}\left(r\right)+\beta \left(P_{A}P_{B}-P_{B}\right)\rho_{n}\left(r\right), & r\in S\\ \rho_{n}\left(r\right)-\gamma P_{B}\rho_{n}\left(r\right), & r\notin S \end{array}\right.\;\left(\mathrm{HDM}\text{-}f6\right) \end{array}$$

A comparative summary of iterative phasing algorithms is provided in Table 1. The six formulas collectively constitute the complete Hybrid Difference Map (HDM) algorithm series. Among them, HDM-f2, HDM-f4, and HDM-f6 incorporate tunable parameters, the relaxation factor β and the negative feedback factor γ, providing essential flexibility for optimizing algorithmic performance across diverse protein structures. In the implementation, the real-space projection operator $P_{A}$ enforces constraints through protein histogram matching [36] and solvent flattening [39], while the Fourier-space projection operator $P_{B}$ imposes the experimental data constraint by replacing the calculated Fourier amplitudes with the observed diffraction amplitudes. From the optimization perspective, phase retrieval seeks the intersection of two constraint sets (real-space support and reciprocal-space amplitude constraints) in a high-dimensional, non-convex landscape, where successful HDM convergence yields a fixed point near the true solution that simultaneously satisfies both constraints.

### 2.3. Phasing Strategies: From Conventional to Genetic Evolution

To systematically enhance the phase retrieval success rate and efficiency of the HDM algorithm, we employed three progressively advanced phasing strategies. All strategies were executed within the same computational framework to ensure comparability of results. The first phasing strategy is the conventional full-resolution scheme. This scheme serves as the baseline method. From the first iteration cycle, all available experimental diffraction amplitudes (i.e., all data in the ‘working set’) are used for the Fourier-space projection constraint ($P_{B}$). This scheme maximizes the use of all measured data but, because it processes phase information from all frequencies simultaneously, the search space is vast and prone to falling into local optima.

The second strategy, termed the resolution-weighted scheme, implements a coarse-to-fine approach analogous to sketching, where a coarse outline precedes detailed features. This is achieved by applying a time-varying weight to the experimental diffraction amplitudes using a Gaussian low-pass filter defined in Equation (13):

(13) $$\begin{array}{c} |F_{\mathrm{obs},w}\left(h\right)|=\exp\left[-2{\left(\pi \sigma_{w}S_{h}\right)}^{2}\right]\cdot \left|F_{\mathrm{obs}}\left(h\right)\right| \end{array}$$

Here, $S_{h}$ is the magnitude of the diffraction vector for reflection h (i.e., $1/d_{h}$), and $\sigma_{w}$ is the filter radius parameter. Over the first few thousand iteration cycles, $\sigma_{w}$ is typically decreased from an initial value less than 1.0 Å down to 0. In the early stages of iteration ($\sigma_{w}$ large), low-resolution data are emphasized, and high-resolution data are effectively suppressed, allowing the algorithm to locate the overall shape and envelope of the protein molecule. As iteration proceeds, $\sigma_{w}$ gradually decreases, allowing higher-resolution diffraction data to be progressively incorporated into the phasing process, thereby gradually delineating the fine internal structure of the molecule, such as secondary structure and side chains. This ‘coarse-to-fine’ strategy effectively reduces the solution space in the initial stage, increasing the probability of finding the correct molecular envelope. Specific details of the resolution-weighted scheme can be found in our previously published paper [33].

The third strategy employs a genetic evolution framework that enhances the resolution-weighted scheme by integrating a population-based genetic algorithm. This approach leverages swarm intelligence and natural selection principles to overcome local optima. The population contains 100 individuals, implemented as independent Message Passing Interface (MPI) processes for parallel computation. Each individual is initialized with independent random phases and executes 100 HDM iterations to establish an initial population with sufficient diversity. Each individual’s ‘chromosome’ is encoded as the electron density values (real numbers) on a 1 Å grid within the asymmetric unit. Individual quality is evaluated using a dynamic fitness function based on $R_{work}$ (Equation (14)):

(14) $$\begin{array}{cc} f_{i} & =\left\{\begin{array}{cc} \frac{R_{\mathrm{thres}}-R_{\mathrm{work},i}}{R_{\mathrm{thres}}-R_{\min}}, & \mathrm{if}\;R_{\mathrm{work},i}<R_{\mathrm{thres}}\\ 0, & \mathrm{otherwise} \end{array}\right. \end{array}$$

Here, the subscript *i* denotes the individual and $R_{\mathrm{thres}}=R_{\mathrm{avg}}+\left(R_{\mathrm{avg}}-R_{\min}\right)$. The threshold $R_{\mathrm{thres}}$ adapts based on the current overall quality (average $R_{work}$, $R_{\mathrm{avg}}$) and the best quality (minimum $R_{work}$, $R_{\min}$) of the population, thereby maintaining diversity in the early stages of evolution and intensifying selection pressure later.

The genetic algorithm operates through three core mechanisms: (a) Selection employs roulette wheel sampling, where the probability of an individual being selected as a parent is proportional to its fitness $f_{i}$. (b) Crossover maintains spatial continuity of density through a multi-segment strategy. Five independent three-dimensional segments within the asymmetric unit are randomly selected, and density values within these segments are exchanged between two parent individuals, with the number of exchanged grid points accounting for half of the total. (c) Mutation randomly selects grid points in an individual with 1% probability and replaces their electron density values with random numbers in the range [0, 1.0] to introduce genetic diversity.

To prevent premature convergence, we score the similarity between each individual and others in the population, penalizing the fitness of overly similar individuals while directly inheriting elite individuals to the next generation. Critically, before any genetic operation, all electron density maps are rotationally and translationally aligned to achieve maximum spatial overlap of the protein mask with the fittest individual, ensuring that crossover operations produce meaningful offspring. Genetic operations are performed once every 100 HDM iterations, balancing the algorithm’s intrinsic convergence momentum with population-based information sharing. Detailed specifications of this genetic evolution strategy are provided in our previously published work [30].

### 2.4. Electron Density Map Quality Assessment Metrics

We employed a multi-dimensional set of quantitative metrics to comprehensively assess the quality of both the phase retrieval process and final results. These metrics were calculated and recorded at each iteration to monitor convergence dynamics. The core indicators $R_{\mathrm{work}}$ and $R_{\mathrm{free}}$ assess the agreement between calculated and experimental data as well as the risk of overfitting, defined by Equations (15) and (16):

(15) $$\begin{array}{cc} R_{\mathrm{work}} & =\frac{\sum_{h\in \mathrm{work}}\left||F_{\mathrm{obs}}\left(h\right)|-\lambda |F_{\mathrm{cal}}\left(h\right)|\right|}{\sum_{h\in \mathrm{work}}\left|F_{\mathrm{obs}}\left(h\right)\right|} \end{array}$$

(16) $$\begin{array}{cc} R_{\mathrm{free}} & =\frac{\sum_{h\in \mathrm{free}}\left||F_{\mathrm{obs}}\left(h\right)|-\lambda |F_{\mathrm{cal}}\left(h\right)|\right|}{\sum_{h\in \mathrm{free}}\left|F_{\mathrm{obs}}\left(h\right)\right|} \end{array}$$

Here, $|F_{\mathrm{obs}}\left(h\right)|$ and $|F_{\mathrm{cal}}\left(h\right)|$ denote the experimentally observed and currently calculated structure factor amplitudes, respectively, while λ represents a scaling factor. Simultaneous decrease of both $R_{\mathrm{work}}$ and $R_{\mathrm{free}}$ provides strong evidence of convergence toward the correct solution.

The mean phase error Δφ directly quantifies the deviation between retrieved phases and reference ‘true’ phases (calculated from the PDB structure) in degrees, as defined by Equation (17):

(17) $$\begin{array}{cc} \Delta \varphi & =\frac{\sum_{h\in \mathrm{work}}\arccos\left(\cos(\varphi_{\mathrm{true}}\left(h\right)-\varphi_{\mathrm{cal}}\left(h\right))\right)}{N_{\mathrm{work}}} \end{array}$$

Here, $N_{\mathrm{work}}$ is the number of unique reflections in the working set. To quantify the spatial overlap accuracy between the *ab initio* reconstructed protein mask ($S_{\mathrm{cal}}$) and the true mask ($S_{\mathrm{true}}$), we employed the intersection over union (IoU) metric, defined by Equation (18):

(18) $$\begin{array}{cc} \mathrm{IoU} & =\frac{|S_{\mathrm{cal}}\cap S_{\mathrm{true}}|}{|S_{\mathrm{cal}}\cup S_{\mathrm{true}}|} \end{array}$$

An IoU value approaching unity indicates more accurate envelope reconstruction.

As an internal convergence criterion independent of known structural information, we monitored regional density deviations by averaging the absolute values of specific density terms over grid points in the protein and solvent regions. For HDM-f1, these deviations are defined by Equation (19):

(19) $$\begin{array}{cc} \Delta \rho & =\left\{\begin{array}{cc} \langle |\;\left(2P_{A}P_{B}-P_{A}-P_{B}\right)\rho \left(r\right)|\rangle , & r\in S\\ \langle |P_{B}\rho \left(r\right)|\rangle , & r\notin S \end{array}\right. \end{array}$$

where 〈·〉 denotes averaging over grid points in each region. Similar definitions apply to the other five HDM formulas. These deviation values typically remain high during the search phase and decrease significantly alongside the R-factors as the iteration converges toward the global optimum.

For reflections that are missing, belong to the free set, exhibit large measurement errors ($\sigma_{|F_{obs}|}>2.0\times \left|F_{obs}\right|$), or fall below approximately 15 Å resolution, the corresponding amplitudes were replaced using calculated values according to Equation (20):

(20) $$|F_{\mathrm{miss}}\left(h\right)|=\frac{\sum_{h^{\prime }\in \mathrm{work}}\left|F_{\mathrm{obs}}\left(h^{\prime }\right)\right|}{\sum_{h^{\prime }\in \mathrm{work}}\left|F_{\mathrm{cal}}\left(h^{\prime }\right)\right|}\;\left|F_{\mathrm{cal}}\left(h\right)\right|$$

### 2.5. Structure Modeling and Validation

For successfully converged electron density maps with low phase error, we performed fully automated atomic model building to verify their utility in practical structure determination. The final MTZ file containing the retrieved phases, along with the protein amino acid sequence, was used as input for automated model building with ARP/wARP (v8.0) [40,41], Buccaneer [42] from the CCP4 software suite (v9) [34], or Phenix (v1.16-3549) AutoBuild [35,43]. These programs automatically identify and place amino acid residues within the electron density map.

The initially built model typically contains over 80% of the amino acid residues. We employed Phenix.refine [37] to perform one round of rigid-body refinement on this model to eliminate minor steric clashes that may arise during automated building. The refined model was then superimposed onto the reference PDB structure using least-squares fitting of backbone atoms (Cα, C, N), and the backbone root-mean-square deviation (RMSD) was calculated. This RMSD serves as the final indicator for assessing atomic-level accuracy of the reconstructed model. An RMSD below 0.5 Å typically indicates that the reconstructed structure achieves high accuracy at the atomic level.

## 3. Results

### 3.1. HDM Algorithm Enables Ab Initio Atomic-Level Structure Reconstruction: A Case Study on 2uxj

To visually demonstrate the capability of the HDM algorithm to recover high-precision electron density maps starting from random phases, we first conducted a detailed analysis using protein 2uxj [44] (a transmembrane photosynthetic reaction center protein-ligand complex from *rhodobacter sphaeroides*, resolution 2.25 Å, estimated solvent content 76%) as an example, under the conventional full-resolution phasing scheme using the HDM-f1 iterative formula. Starting from 100 sets of random phases, each set independently underwent 10,000 iterations, with six key metrics monitored in real-time to track the convergence process.

**Figure 2:**
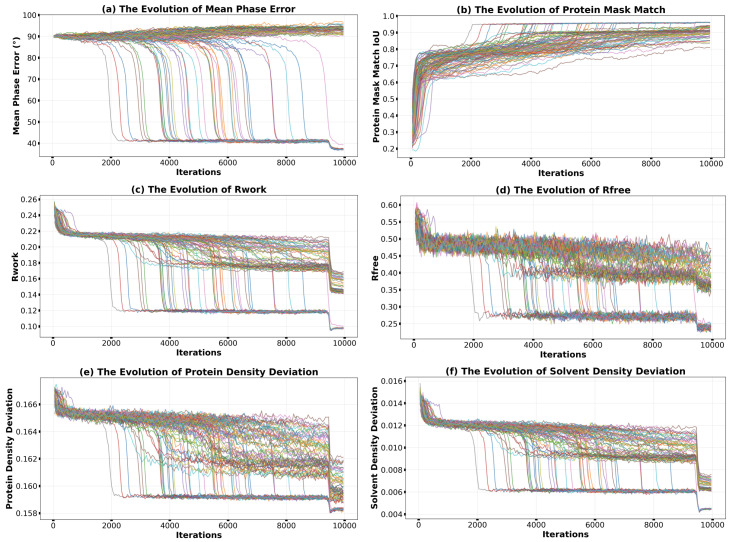
Convergence monitoring for HDM-f1 phasing of 2uxj using the conventional full-resolution scheme. Evolution of (**a**) mean phase error, (**b**) protein mask match (IoU), (**c**) $R_{work}$, (**d**) $R_{free}$, (**e**) protein density deviation, and (**f**) solvent density deviation over 10,000 iterations for 100 independent trials. The sudden improvements in all metrics indicate successful convergence events.

As shown in Figure 2, all monitoring metrics clearly delineate the dynamic trajectory of phase retrieval. In the early stages of iteration (approximately the first 2000 iterations), the mean phase error (Figure 2a) remained around 90°, the protein envelope match IoU (Figure 2b) was about 0.8, and $R_{work}$, $R_{free}$ (Figure 2c,d), as well as the density deviations in the protein and solvent regions (Figure 2e,f), were all at high levels, indicating that the system was exploring a vast solution space. After the 2000th iteration, a successful reconstruction individual exhibited a convergence phase transition: its phase error suddenly dropped from 90° to around 40° within a short period, while the IoU jumped above 0.9, and $R_{work}$ and $R_{free}$ also decreased significantly. Particularly important is that the internal criteria, which do not rely on known structural information—the absolute values of the relaxation density in the protein region and the negative feedback density in the solvent region—also showed a significant decrease. This strongly proves that the convergence was not overfitting but indeed approached the global optimum. Notably, some traces in Figure 2c,d drop to intermediate *R*-factor values between the successful and failed runs, indicating iterations trapped in local minima. Analysis reveals that these cases typically exhibit correct molecular envelopes but with partially inverted internal densities—a consequence of the fact that density inversion (ρ→−ρ) preserves diffraction amplitudes, and histogram matching alone is sometimes insufficient to resolve this ambiguity. In the last 500 iterations, HDM was gradually turned off, and solvent flattening was applied, reducing the phase error further to around 37°. Ultimately, 41 out of 100 independent runs successfully converged, yielding a success rate of 41%. These calculations were completed on a Dell R740 server equipped with 52 cores (104 threads) operating at 2.1 GHz. The entire computation, comprising 100 independent trials of 10,000 iterations each starting from random phases, required approximately 3 h.

**Figure 3:**
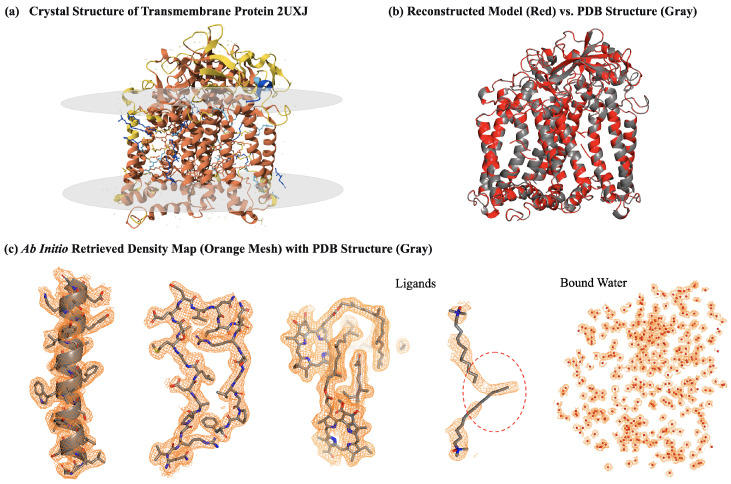
Results of *ab initio* structure determination for 2uxj using HDM-f1. (**a**) The structure of a transmembrane photosynthetic reaction center protein-ligand complex (PDB: 2uxj) from *rhodobacter sphaeroides*. (**b**) Superposition of the *ab initio* built model (red) and the PDB-deposited structure (gray), showing high agreement (backbone RMSD = 0.15 Å). (**c**) The reconstructed electron density map (orange mesh) contoured around the PDB-deposited structure (gray sticks). The red circle indicates the position of a ligand which appears inconsistent with the *ab initio* calculated density map, visually demonstrating the capability of direct phasing to validate model accuracy and identify potential structural discrepancies. Structures and density maps were visualized using PyMOL 3.1 [45].

Alignment and averaging of the successfully converged electron density maps yielded a final, high-quality electron density map. The averaged map exhibits exceptional quality, featuring clear and continuous density for the protein backbone and most side chains, which indicates strong interpretability. Based on this map, fully automated model building with ARP/wARP (v8.0) produced an atomic model (Figure 3b) that is in excellent agreement with the PDB-deposited reference structure, achieving a backbone atom RMSD of approximately 0.15 Å and a sequence coverage of 97%. Subsequent one round of rigid-body refinement further improved the model quality, reducing the model calculated $R_{\mathrm{work}}$ and $R_{\mathrm{free}}$ values from 0.258 to 0.241 and from 0.260 to 0.259, respectively, while the backbone RMSD decreased to 0.10 Å. This result robustly demonstrates that the HDM-f1 algorithm possesses the capability for *ab initio* reconstruction of protein structures with atomic-level accuracy directly from random phases.

Figure 3c provides a direct visual comparison of the electron density and the PDB deposited model. Notably, well-ordered solvent molecules on the protein surface become distinctly visible, and the density for ligands bound at the polymer–membrane interface is clearly defined. Furthermore, the *ab initio* map reveals potential local errors in the deposited model, as indicated by a red circle in Figure 3c, where the position of one of the ligands appears inconsistent with the *ab initio* calculated density. This discrepancy was consistently observed across multiple independent reconstructions starting from different random phases, ruling out artifacts from individual runs. The affected ligands are located close to the molecular surface with high temperature factors (60–80 ${Å}^{2}$) in the deposited model, suggesting room for refinement improvement. This observation highlights a key strength of direct phasing methods: their model-free nature grants them the potential to identify and rectify structural errors that may arise from AI prediction biases or inaccuracies introduced by molecular replacement.

### 3.2. Synergistic Advancement of Phasing Strategies: Resolution Weighting and Genetic Algorithm Significantly Enhance HDM Performance

Although the conventional phasing scheme can successfully reconstruct structures, its success rate and convergence speed remain limited. To address these limitations, we integrated the HDM-f1 algorithm with more advanced strategies, employing the same test case (2uxj) to evaluate the resulting performance gains.

The introduction of the resolution-weighted scheme [33] led to the first notable improvement. By prioritizing low-resolution data to first establish the molecular envelope, this strategy optimizes the search path. Compared to the conventional full-resolution approach, the resolution-weighted scheme typically yields a slightly higher success rate, and the convergence trajectory is smoother, indicating more stable guidance toward the correct solution.

**Figure 4:**
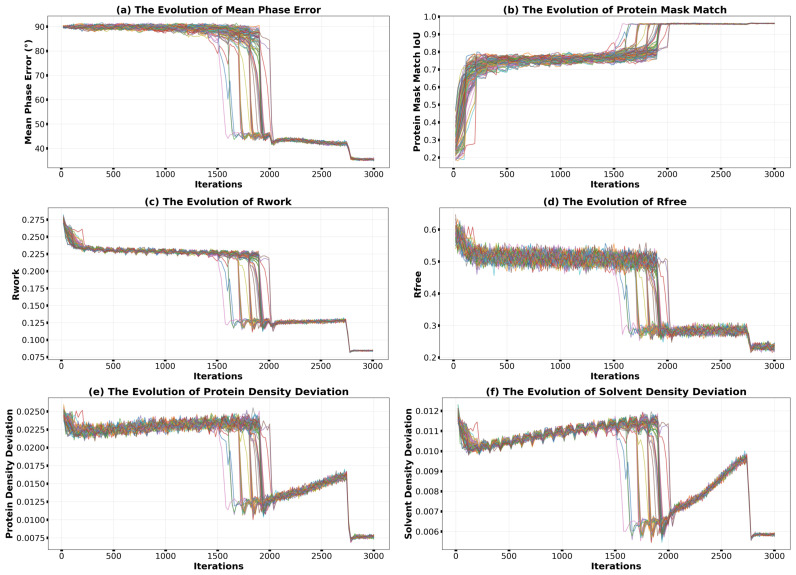
Performance enhancement using the genetic evolution scheme combined with resolution weighting for HDM-f1 on 2uxj. Evolution of (**a**) mean phase error, (**b**) protein mask match (IoU), (**c**) $R_{work}$, (**d**) $R_{free}$, (**e**) protein density deviation, and (**f**) solvent density deviation. Note the ‘collective’ convergence of the entire population of 100 trials after the first individual converges, leading to a 100% success rate.

Building upon this, the addition of the genetic evolution scheme [30] delivered a substantial performance leap. As illustrated in Figure 4 (in contrast to Figure 2), the genetic algorithm leverages the principle of population co-evolution. At approximately 1500 iterations, the first converging individual emerges within the population, accompanied by abrupt improvements in all monitoring metrics. Through subsequent selection and crossover operations, the advantageous traits manifested as correct electron density features of this individual are rapidly propagated across the population. Notably, the crossover mechanism can rescue iterations trapped in local minima (such as those with correct envelopes but inverted densities) by combining favorable density features from different individuals. Consequently, all 100 individuals achieve successful convergence within the following 500 iterations, as evidenced by the collective trajectories converging to a low phase-error region in Figure 4a. This characteristic population-wide convergence pattern elevates the final success rate to 100%, a dramatic increase from the 41% achieved under the conventional scheme. Under the genetic evolution scheme, once all 100 trials successfully converge, the algorithm terminates early and concludes with solvent flattening.

Beyond boosting the success rate, the genetic evolution scheme also markedly accelerated the overall convergence. The median number of iterations required for convergence was significantly reduced. Furthermore, the availability of multiple, independently converged solutions from the population enabled effective reduction of random errors through averaging. For the 2uxj test case, this process further refined the final mean phase error from approximately 34° (after solvent flattening) to around 28°, yielding electron density maps of superior quality for downstream modeling. It is worth noting that in Figure 4e,f, the density deviations increase after convergence. This behavior arises from the resolution-weighting scheme employed in conjunction with the genetic algorithm: during early iterations, primarily low-resolution data contribute to the reconstruction, resulting in smoother density maps with smaller deviations; as high-resolution data are progressively incorporated, the density deviations naturally increase due to the finer structural details being resolved. Control experiments using the genetic algorithm alone (without resolution weighting) confirmed that the density deviations remain essentially constant after convergence. Regarding computational cost, although the genetic operations are relatively time-consuming, they are applied only once every 100 HDM iterations; combined with early termination triggered when all individuals converge, the genetic evolution scheme combined with resolution weighting typically adds less than one hour to the total runtime compared to the conventional scheme.

In summary, the progression from the conventional scheme to resolution weighting and finally to genetic evolution delineates a clear hierarchy of performance enhancement. Each strategy contributes to progressively improving the success rate and ultimate accuracy of the HDM algorithm, with the genetic evolution scheme additionally achieving substantial gains in convergence speed, thereby constituting a powerful, combined methodology for tackling challenging phasing problems.

### 3.3. Systematic Performance Evaluation: HDM Algorithms Outperform Traditional DiffMap Under Multiple Strategies

To comprehensively assess the universality and performance of the HDM algorithm series, we conducted systematic evaluation across 22 diverse protein structures. Eight iterative algorithms, comprising the six HDM formulas, HIO, and DiffMap, were tested under each of three distinct phasing schemes. For every combination of structure, algorithm, and scheme, 100 independent trials were performed, each executing 10,000 iterations from random starting phases. In all tests involving the parameterized HDM formulas (HDM-f2, HDM-f4, and HDM-f6), the relaxation factor β and negative feedback factor γ were fixed at β=γ=0.75.

**Figure 5:**
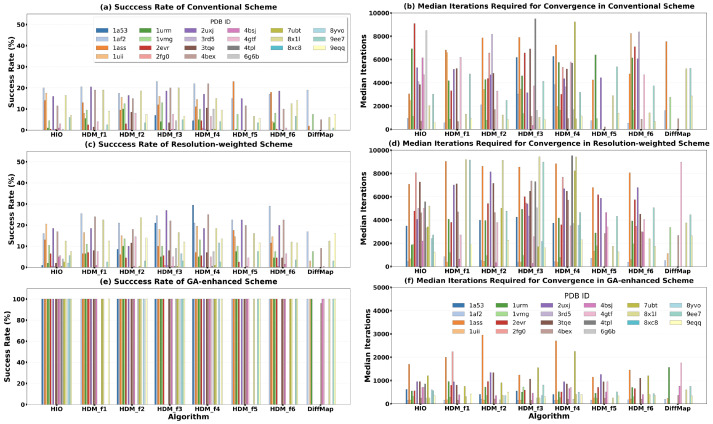
Performance comparison of eight iterative algorithms (6 HDM formulas, HIO, DiffMap) across three phasing schemes on 22 protein structures. (**a**,**c**,**e**) Success rates and (**b**,**d**,**f**) median iterations to convergence under the (**a**,**b**) conventional, (**c**,**d**) resolution-weighted, and (**e**,**f**) genetic evolution schemes. Each bar group represents one algorithm tested on 22 structures.

**Figure 6:**
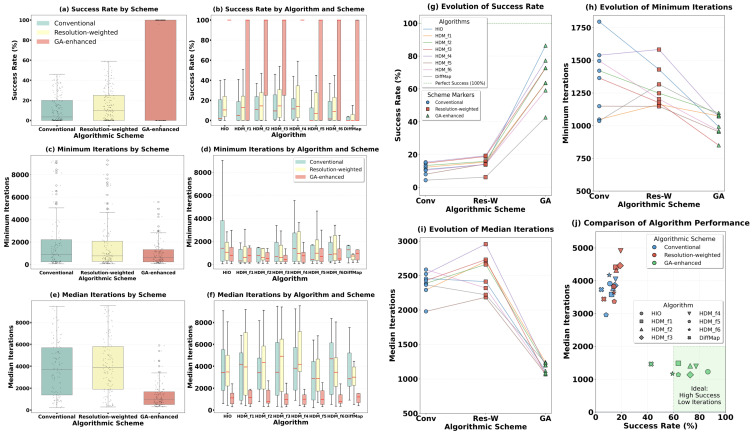
Statistical summary of algorithm performance. (**a**,**c**,**e**) Box plots showing the distribution of (**a**) success rate, (**c**) minimum iterations, and (**e**) median iterations across all 22 structures for each of the three schemes (conventional, resolution-weighted, GA-enhanced). (**b**,**d**,**f**) Box plots showing the same metrics for each of the eight algorithms under the three schemes. (**g**–**i**) Line plots showing the evolution of (**g**) success rate, (**h**) minimum iterations, and (**i**) median iterations across the three schemes for the eight algorithms. (**j**) Scatter plot of success rate versus median iterations to convergence, highlighting the high-performance cluster of HDM algorithms and HIO under the GA-enhanced scheme.

Figure 5 and Figure 6 comprehensively display the phase retrieval success rates and convergence speeds of these eight algorithms under the three schemes, with detailed numerical results provided in Appendix A, Table A2, Table A3 and Table A4. The three schemes exhibit a clear performance hierarchy. Under the conventional full-resolution scheme (Figure 5a,b), the six HDM iterative formulas demonstrated varying potential, with their average success rates obviously higher than that of the traditional DiffMap algorithm. Notably, in Figure 5a, the average success rates of HDM-f1, HDM-f2, HDM-f3, and HDM-f4 surpassed that of the well-established HIO algorithm, while HDM-f5 and HDM-f6 were slightly inferior to HIO.

When the resolution-weighted scheme was introduced (Figure 5c,d), the success rates of all algorithms showed a slight improvement. This indicates that the ‘coarse-to-fine’ phasing logic has a universally positive effect on various iterative algorithms. However, the median number of iterations required for successful convergence under this scheme increased slightly, possibly because the gradual introduction of high-resolution data prolonged the refinement process of the density map.

The most breakthrough performance enhancement came from the genetic evolution scheme (Figure 5e,f). Under this scheme, the success rates and convergence speeds of almost all algorithms (including DiffMap) improved qualitatively. The success rates of the six HDM formulas and HIO reached or approached 100%, and the median number of iterations required for successful convergence was reduced by more than half compared to the conventional scheme. This indicates that the genetic algorithm effectively compensates for the inherent limitations in the global search capability of individual algorithms through population information sharing.

The statistical box plots, line plots and scatter plot in Figure 6 further reveal the overall performance of different schemes and algorithms. Figure 6a clearly shows that from the conventional scheme to resolution weighting, and then to genetic evolution, the three schemes brought about progressive improvements in success rate. For convergence speed, the resolution-weighted scheme showed comparable minimum iterations (Figure 6c) but slightly increased median iterations (Figure 6e) relative to the conventional scheme, while the genetic evolution scheme achieved substantial reductions in both metrics. The same trends are observed for each individual iterative algorithm under the three schemes, as shown in Figure 6b,d,f. Figure 6g–i visually demonstrate these performance trends for the eight algorithms using line plots. The scatter plot in Figure 6j provides a final comprehensive comparison: under the highest-performing genetic evolution scheme, the six HDM iterative formulas and HIO cluster in the ‘high-performance region’ characterized by high success rate and low iteration count (average success rate across different structures > 60%, median number of iterations required for successful convergence < 2000). Their overall performance is significantly superior to the traditional DiffMap algorithm.

In summary, systematic testing demonstrates that the HDM series algorithms are a class of efficient and reliable phase retrieval methods. Their overall performance is on par with the mature HIO algorithm and significantly surpasses the traditional DiffMap algorithm. When combined with the genetic evolution scheme, their performance can reach a near-optimal state.

### 3.4. The Critical Role of Solvent Content and Its Interaction with Phasing Strategies

The success of iterative projection algorithms is generally closely related to the solvent content of the crystal [22,28,30]. We further analyzed the relationship between the phase retrieval success rate of the 22 test structures and their respective solvent contents under the three different phasing strategies.

**Figure 7:**
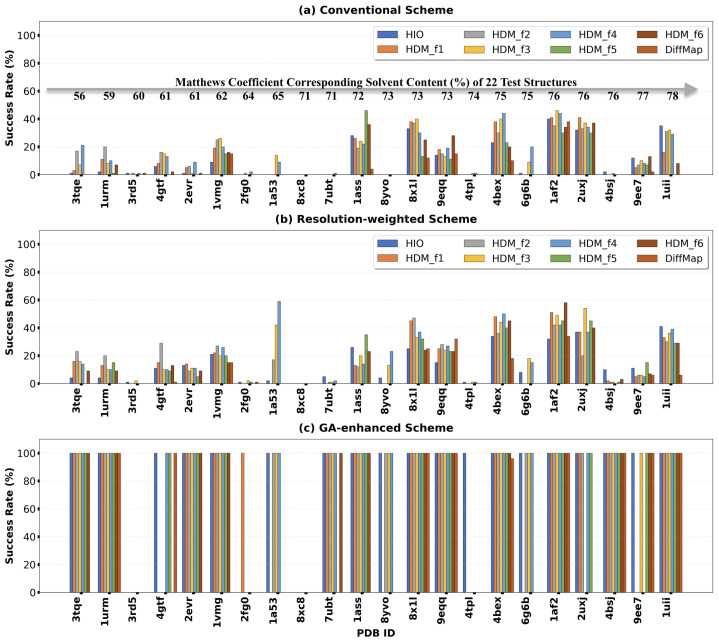
Success rates of the eight iterative algorithms on the 22 test structures under the (**a**) conventional, (**b**) resolution-weighted, and (**c**) genetic evolution schemes. Each bar group corresponds to one structure, with the eight colored bars representing the eight different algorithms. From left to right, structures are arranged in ascending order of solvent content as estimated by the Matthews coefficient.

As shown in Figure 7a, under the conventional full-resolution scheme, the success rate generally increases with solvent content, though with considerable variation among structures. Structures with high solvent content (e.g., >70%), such as 1ass [46], 8x1l [47], 9eqq [48], 4bex [49], 1af2 [50], 2uxj [44], 9ee7 [51] and 1uii [52], generally achieved high success rates. In contrast, when the solvent content approached or fell below 60% (e.g., 3rd5 [53], 2fg0 [54]), the success rate dropped sharply or even to zero. This phenomenon is consistent with traditional understanding, as higher solvent content means the ‘solvent flat’ constraint occupies a larger proportion in real space, thus more effectively driving iterative convergence [17,55]. Beyond solvent content, several factors increase the difficulty of *ab initio* molecular envelope determination. First, abundant bound water molecules on the protein surface must be enclosed within the reconstructed envelope, effectively reducing the solvent region available for the flatness constraint; this is particularly problematic for structures such as 3rd5 (413 water molecules, 16% of non-hydrogen atoms), 2fg0 (428, 11%), 7ubt [56] (439, 9%), and 8yvo [57] (350, 10%). Second, space group symmetry and enantiomorphic ambiguity in non-centrosymmetric space groups give rise to multiple equivalent origin choices, where different origins can yield nearly indistinguishable molecular envelopes, hindering convergence to a unique solution (e.g., 3rd5, 2fg0, 8xc8 [58], 8yvo, 4tpl [59]). Third, highly interdigitated protein and solvent regions create complex boundaries that impede accurate *ab initio* envelope reconstruction (e.g., 7ubt, 4tpl, 4bsj [60]). Additionally, low-resolution reflections with poor signal-to-noise ratios or large measurement errors further compromise phase retrieval quality. These challenges are particularly pronounced in crystals with limited solvent content, making successful phase recovery difficult to achieve.

**Figure 8:**
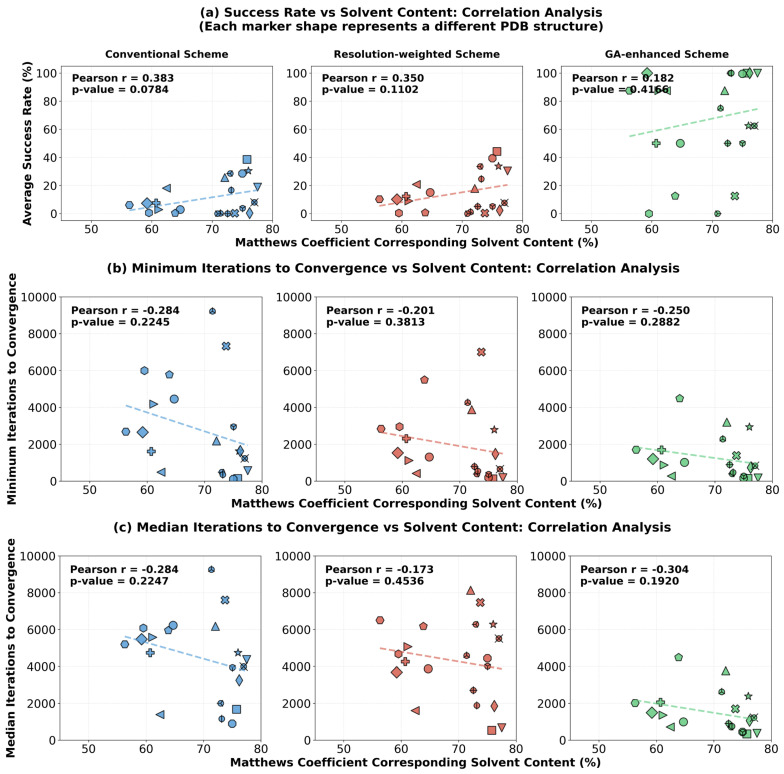
Correlation analysis between solvent content and phase retrieval performance metrics under the three phasing schemes (conventional, resolution-weighted, GA-enhanced). (**a**) Success rate vs. solvent content. (**b**) Minimum iterations to convergence vs. solvent content. (**c**) Median iterations to convergence vs. solvent content. Each marker represents a PDB structure. The Pearson correlation coefficient (r) between success rate and solvent content, along with its associated *p*-value, is indicated for each subplot.

When the resolution-weighted scheme and the genetic evolution scheme were introduced, although the overall success rates increased substantially, the correlation between success rate and solvent content weakened accordingly (Pearson *r* decreasing from 0.383 to 0.182, Figure 8a). The scatter plot for the genetic evolution scheme shows that even for some structures with solvent content near 60%, the success rate was boosted to 100%. This indicates that advanced phasing strategies can, to a considerable extent, overcome the inherent difficulties posed by lower solvent content, thereby broadening the applicability range of direct phasing methods. The analysis of convergence speed (Figure 8b,c) shows weak negative correlations between solvent content and both minimum iterations and median iterations across all three schemes, suggesting that higher solvent content tends to facilitate faster convergence.

In summary, crystal solvent content is a fundamental key factor affecting the success rate of direct phasing. However, advanced strategies like resolution weighting and genetic evolution, particularly the latter, can effectively alleviate the constraint weakening caused by lower solvent content through optimized search paths and swarm intelligence. This significantly enhances the success probability for more challenging structures, thereby practically expanding the applicable boundaries of direct phasing methods.

### 3.5. Density Averaging: An Effective Post-Processing Step to Enhance Phase Accuracy and Map Quality

A direct benefit of the high phase retrieval success rate is the ability to obtain multiple independently converged, approximately correct electron density maps. We explored the strategy of aligning and averaging multiple successful solutions as a post-processing step to further improve the map quality. Generally, averaging a larger number of successful solutions leads to reduced phase errors, but with diminishing returns. When more than 20 solutions are averaged, the additional improvement becomes marginal.

**Figure 9:**
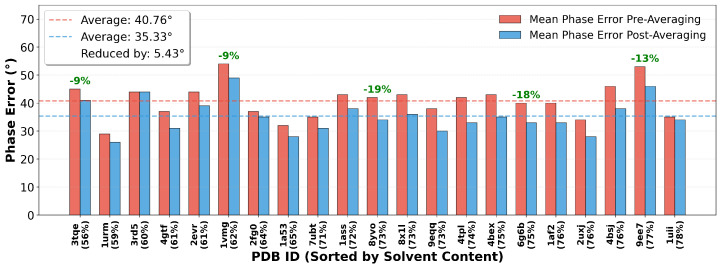
Improvement in phase accuracy through averaging of successfully converged density maps. Comparison of mean phase error for each of the test structures before (red) and after (blue) averaging about 100 successfully converged maps. Structure 8xc8 is excluded due to zero success rate, and 3rd5 shows no improvement due to only one successful convergence. The overall average phase error across the remaining 21 structures is reduced from 40.76° to 35.33°, a reduction of 5.43°.

As shown in Figure 9, we calculated the mean phase error before and after averaging over 100 successfully converged density maps under GA-enhanced scheme across test structures. The results indicate that density averaging can universally and stably reduce the phase error. For all structures, wherever multiple (≥2) successful convergence results existed, the phase error after averaging was lower than before averaging. Overall statistics show that the mean phase error was significantly reduced from 40.76° before averaging to 35.33° after averaging, with an average reduction of 5.43°. This improvement is statistically significant and brings the final phase error into a range more favorable for model building.

Notably, the reduction in phase error directly translates into a visible improvement in electron density map quality. The averaged maps exhibit a higher signal-to-noise ratio, with more continuous and clear density for the protein backbone and side chains, and effectively suppressed noise in the background regions. This enhanced map quality significantly reduces the difficulty of automated model building and holds the promise of constructing more complete atomic models. One exceptional case is structure 3rd5 (Figure 9), where the phase error remained unchanged after averaging. This is because predominantly only one successful convergence result was obtained for this structure during testing, making effective averaging impossible. This conversely confirms that a high success rate is a prerequisite for implementing effective density averaging.

In summary, density averaging is a simple yet powerful post-processing technique. By leveraging the multiple approximate solutions provided by the high success rate of the HDM algorithm, it reduces random error through ensemble averaging, consistently lowering the phase error by approximately 5.43°. This yields higher-quality, more interpretable electron density maps, laying a more solid foundation for subsequent automated model building.

### 3.6. Parameter Sensitivity Analysis: Optimization Space for Relaxation and Negative Feedback Factors

**Figure 10:**
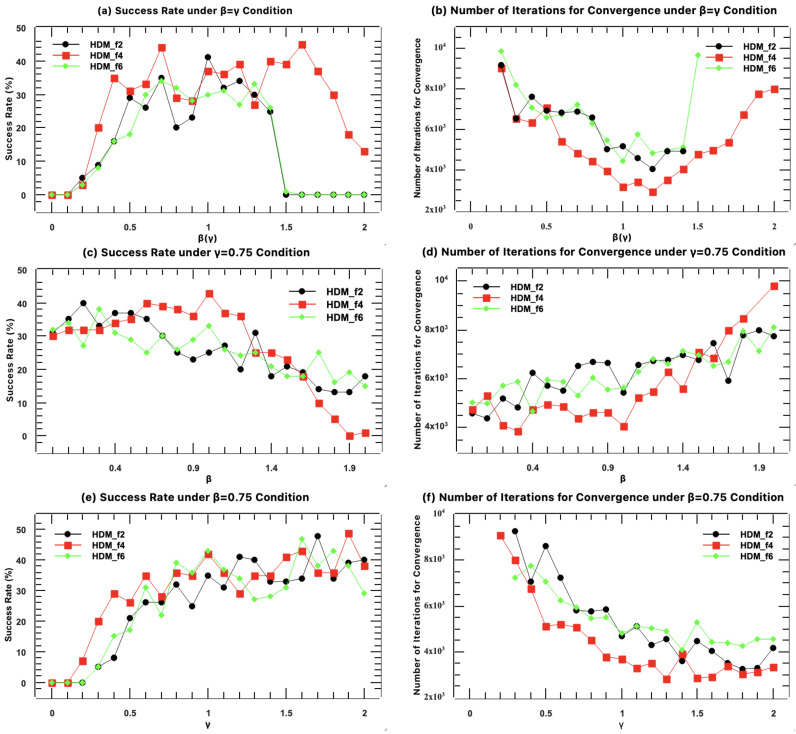
Parameter sensitivity analysis for HDM-f2, f4, and f6 on structure 2uxj. (**a**) Success rate and (**b**) median iterations to convergence as functions of the feedback parameters when β=γ. (**c**) Success rate and (**d**) median iterations as functions of the relaxation factor β with γ fixed at 0.75. (**e**) Success rate and (**f**) median iterations as functions of the negative feedback factor γ with β fixed at 0.75. Each data point represents the success rate, derived from 100 independent trials (10,000 iterations each under the conventional full-resolution scheme) starting from random phases.

Among the HDM series algorithms, HDM-f2, HDM-f4, and HDM-f6 contain tunable relaxation (β) and negative feedback (γ) factors. To determine the optimal operational range for these key parameters, we systematically analyzed their impact on algorithm performance using 2uxj as an example. In Figure 10, each data point represents the result of 100 independent trials starting from random phases, using the conventional full-resolution scheme with 10,000 iterations each, from which the probability of successful phase recovery was calculated.

We first varied β and γ synchronously (β=γ). As shown in Figure 10a,b, as the parameter value increased from 0, the success rate rose rapidly and remained high within the range of 0.5 to 1.2; simultaneously, the median number of iterations required for convergence decreased, indicating that moderate parameter increase aids in accelerating convergence. However, when the parameter value exceeded 1.3, the success rate dropped sharply to zero, and the iteration count soared, indicating that excessively strong feedback disrupts algorithm stability.

To decouple the effects of the two parameters, we performed tests fixing one parameter at a time. When the negative feedback factor γ was fixed at 0.75 and only the relaxation factor β was varied (Figure 10c,d), we found that even with β=0 (i.e., no relaxation term in the protein region), the success rate remained considerable. This indicates that the negative feedback in the solvent region is the primary driving force for algorithm convergence. As β increased from 0 to 1.0, the success rate remained stable or increased slightly, while the median iterations required for convergence remained stable or increased slowly.

Conversely, when the relaxation factor β was fixed at 0.75 and only the negative feedback factor γ was varied (Figure 10e,f), the results were particularly striking: when γ=0, the success rate was zero. This strongly proves that the negative feedback mechanism is an indispensable core component for the success of the HDM algorithm. As γ increased from 0 to 1.0, the success rate climbed sharply, and the median iterations required for convergence decreased synchronously.

In summary, parameter analysis reveals the distinct roles of the two factors in the HDM algorithm: the negative feedback factor (γ) is the decisive parameter determining whether the algorithm succeeds, while the relaxation factor (β) primarily plays an auxiliary fine-tuning role. Based on these results, we recommend setting the values of β and γ within the range of 0.5 to 1.0. Within this range, the algorithm maintains high success rate, relatively fast convergence speed, and stable operation. This provides clear and flexible guidance for users when setting parameters in practical applications.

## 4. Discussion

The results presented above demonstrate that the Hybrid Difference Map algorithm achieves robust phase retrieval performance across diverse protein structures. In the following sections, we discuss the underlying factors contributing to this success, the practical implications of our findings, and the remaining challenges for future development.

### 4.1. Advantages and Universality of the HDM Algorithm

The results clearly demonstrate that the six HDM iterative formulas constitute a high-performance algorithm family. Compared to DiffMap, the success rate of HDM more than doubled (Figure 5 and Figure 6). This improvement stems from its more thorough utilization of dual-space constraints between real and reciprocal space. In the protein region, the optimized relaxation term more effectively prevents iterations from stagnating in local minima. In the solvent region, the negative feedback term borrowed from HIO provides a powerful, continuous driving force for convergence. It is noteworthy that formulas such as HDM-f1, HDM-f2, HDM-f3, and HDM-f4 even demonstrated potential comparable to or superior to HIO in some tests under the conventional full-resolution scheme (Figure 5a), providing justification for trying new algorithms on specific hard-to-solve structures. Therefore, when HIO or DiffMap alone proves ineffective, the HDM series algorithms offer multiple efficient alternative options, increasing the probability of solving the phase problem.

An important observation is that no single HDM variant or HIO consistently outperforms all others across all structures. This is expected because HIO, and all HDM variants fundamentally employ the same physical constraints (solvent flatness, histogram matching, and diffraction amplitudes), differing only in how these constraints are applied during iteration. Consequently, they represent variations within the same algorithmic family. However, the practical value of the HDM series lies precisely in this diversity: for a challenging structure where one algorithm yields near-zero success rate—potentially requiring thousands of random starting phases to obtain a single solution—an alternative HDM formula may exhibit a moderately higher success rate, enabling successful phasing with only hundreds of trials. Thus, the six HDM formulas substantially expand the algorithmic toolkit and improve the overall probability of solving difficult structures.

### 4.2. Synergistic Effects of Phasing Strategies and the Mechanism of the Genetic Algorithm

Our research further reveals a profound synergistic effect between the iterative algorithms and the phasing strategies. The three schemes, conventional, resolution-weighted, and genetic evolution, form a stepwise improvement of progressively enhancing performance. The resolution-weighted scheme implements a coarse-to-fine strategy by prioritizing the recovery of molecular envelopes from low-resolution data before resolving high-resolution details. The introduction of the genetic evolution scheme, however, brings a qualitative leap. It incorporates swarm intelligence into the complex global optimization problem of phase retrieval. Once a converging individual appears in the population, its correct ‘density pattern’ can be rapidly disseminated throughout the population via selection and crossover operations, ultimately guiding the entire group to achieve coordinated convergence (Figure 4). This mechanism pushes the success rate close to 100% and halves the median number of iterations required for convergence compared to the conventional scheme. However, it must be emphasized that the success of the genetic algorithm presupposes the emergence of at least one converging individual within the population. The algorithm itself does not completely overcome the physical constraints determined by factors like solvent content but rather significantly improves the efficiency and probability of approaching and locking onto the global optimum solution.

### 4.3. Alleviation of Solvent Content Constraints and the Value of Density Averaging

This study reaffirms that crystal solvent content is a fundamental physical factor limiting the success of direct phasing (Figure 7 and Figure 8). However, a significant finding is that advanced phasing strategies, particularly the genetic evolution scheme, can significantly mitigate the adverse effects associated with moderate solvent content (Figure 8a). This implies that our method practically expands the applicable boundaries of direct phasing methods, making some structures with solvent content near 60%, previously considered difficult to solve by traditional direct methods, viable targets. Furthermore, a direct and valuable byproduct of the high success rate is the ability to average multiple independently converged solutions. Our results show that this simple post-processing operation can stably reduce the phase error by approximately 5.43° (Figure 9), thereby yielding electron density maps with a higher signal-to-noise ratio that are easier to interpret and model. This is not merely a numerical improvement but a significant enhancement to the practicality of the structure determination pipeline.

### 4.4. Parameter Robustness and Practical Application

The parameter analysis of the relaxation factor (β) and negative feedback factor (γ) in HDM-f2, f4, and f6 (Figure 10) provides clear application guidance. The results indicate that γ is the decisive parameter for algorithmic success, while β primarily plays an auxiliary regulatory role. Both parameters maintain good algorithm performance within a broad range of 0.5 to 1.0. This lowers the barrier to using the HDM algorithm and enhances its robustness and user-friendliness in practical applications.

### 4.5. Limitations and Future Perspectives

Despite the significant progress achieved by the HDM algorithm, its application remains primarily confined to crystals with high solvent content (>60%). For structures with lower solvent content or greater complexity, future work needs to introduce stronger constraints, such as non-crystallographic symmetry averaging or integrating information from AlphaFold-predicted structures. Another promising direction is the development of dynamic hybrid algorithms that allow adaptive switching between different HDM formulas during a single reconstruction process based on iteration progress, or permitting the coexistence and hybridization of different algorithm individuals within the genetic algorithm population, thereby combining the strengths of various approaches.

## 5. Conclusions

This study successfully developed and systematically validated a novel iterative phase retrieval algorithm named the Hybrid Difference Map (HDM). Through a partition-optimized strategy, the HDM algorithm creatively integrates the relaxation advantages of the Difference Map algorithm in the protein region with the efficient negative feedback mechanism of the Hybrid Input–Output algorithm in the solvent region. The six iterative formulas derived from this fusion significantly enrich the toolbox for direct phasing methods.

Through extensive testing on 22 protein structures spanning various space groups and resolutions, we confirmed that the HDM series algorithms significantly outperform the traditional DiffMap algorithm in both phase retrieval success rate and convergence speed, achieving an overall performance level comparable to the mature HIO algorithm. Further investigation demonstrated that combining the HDM algorithm with our previously proposed resolution-weighted scheme and genetic evolution scheme constructs a performance hierarchy of phasing schemes. The genetic evolution scheme, leveraging population synergy, elevates the success rate to nearly 100%, reduces the median number of iterations required for convergence by more than half, and effectively broadens the applicability of the method to crystal structures with solvent content near 60%.

Furthermore, the high success rate enables the averaging of multiple independently converged results. This post-processing step stably reduces the phase error by approximately 5°, consequently producing higher-quality electron density maps that are more amenable to automated model building. Parameter sensitivity analysis further confirms the robust performance of the HDM algorithm across a wide parameter range, facilitating its practical application.

In summary, the HDM algorithm and its combination with advanced phasing strategies, as presented in this work, provide a more powerful, efficient, and reliable solution suite for direct methods in protein crystallography. This not only offers a new technical pathway for the *ab initio* determination of unknown protein structures but also provides a robust tool for addressing important scientific questions such as the validation of AI-predicted structures. The compiled algorithms developed in this study are available on GitHub (https://github.com) [61].

## Figures and Tables

**Table 1:** Summary of iterative phasing algorithms. The projection operators $P_{A}$ (real-space) and $P_{B}$ (Fourier-space) enforce support/histogram and amplitude constraints, respectively.

Algorithm	Protein Region	Solvent Region	Parameters	Performance *^a^*
DiffMap *^b^*	$\rho_{n}+\left(2P_{A}P_{B}-P_{A}-P_{B}\right)\rho_{n}$	$\rho_{n}-P_{B}\rho_{n}$	$\beta_{0}$	Baseline (1.0×)
HIO	$P_{A}P_{B}\rho_{n}$	$\rho_{n}-\gamma P_{B}\rho_{n}$	γ	2.5×
HDM-f1	$P_{A}P_{B}\rho_{n}+\left(2P_{A}P_{B}-P_{A}-P_{B}\right)\rho_{n}$	$\rho_{n}-P_{B}\rho_{n}$	None	2.8×
HDM-f2	$P_{A}P_{B}\rho_{n}+\beta \left(2P_{A}P_{B}-P_{A}-P_{B}\right)\rho_{n}$	$\rho_{n}-\gamma P_{B}\rho_{n}$	β, γ	3.1×
HDM-f3	$P_{A}P_{B}\rho_{n}+\left(P_{A}P_{B}-P_{A}\right)\rho_{n}$	$\rho_{n}-P_{B}\rho_{n}$	None	3.4×
HDM-f4	$P_{A}P_{B}\rho_{n}+\beta \left(P_{A}P_{B}-P_{A}\right)\rho_{n}$	$\rho_{n}-\gamma P_{B}\rho_{n}$	β, γ	3.5×
HDM-f5	$P_{A}P_{B}\rho_{n}+\left(P_{A}P_{B}-P_{B}\right)\rho_{n}$	$\rho_{n}-P_{B}\rho_{n}$	None	1.8×
HDM-f6	$P_{A}P_{B}\rho_{n}+\beta \left(P_{A}P_{B}-P_{B}\right)\rho_{n}$	$\rho_{n}-\gamma P_{B}\rho_{n}$	β, γ	2.4×

*^a^* Performance trend indicates the average success rate ratio relative to DiffMap across 22 protein structures under the conventional phasing scheme (Appendix A, Table A2), with β=γ=0.75. All HDM variants (1.8–3.5×, mean 2.8×) and HIO (2.5×) substantially outperform the DiffMap baseline. *^b^* The DiffMap formula shown corresponds to the special case $\beta_{0}=1$ for direct comparison with HDM variants.

## Data Availability

The diffraction data were downloaded from the Protein Data Bank at https://www.rcsb.org (accessed on 1 December 2025).

## Appendix A. Structure Information and Numerical Results

**Table A1:** Structure information, data statistics, and *ab initio* phasing results for 22 test cases.

PDB ID	Description	Space Group	Diffraction Resolution	PDB Reported R-Factor	Number of Non-Hydrogen Atoms	Number of Water Molecules	Number of Reflections	Matthews Coefficient Corresponding Solvent Content (%)	Volume Not Occupied by Model (%)	PDB Posted Solvent Content (%)	Δφ Before Averaging Multi Solutions (°)	Δφ After Averaging Multi Solutions (°)
1a53	Indole-glycerol Phosphate Synthase	$P2_{1}2_{1}2_{1}$	2.0	0.159	2264	242	27,283	64.7	58.0	68.5	32	28
1af2	Cytidine Deaminase	$P3_{1}21$	2.3	0.195	2287	49	24,137	75.76	71.2	76.29	40	33
1ass	Thermosome Apical Domain	$P3_{1}21$	2.3	0.222	1226	35	12,774	72.07	66.8	70.0	43	38
1uii	Geminin Coiled-coil Domain	$P2_{1}2_{1}2_{1}$	2.0	0.216	1154	120	20,158	77.5	73.5	65.3	35	34
1urm	Human Peroxiredoxin 5	$P4_{1}2_{1}2$	1.7	0.146	1545	285	28,558	59.2	51.5	63.5	29	26
1vmg	Nucleotide Pyrophosphohydrolase	$I4_{1}22$	1.46	0.142	826	107	24,059	62.4	53.5	62.64	54	49
2evr	Glutamyl-diamino Endopeptidase	$P4_{1}22$	1.6	0.159	2148	300	48,970	61.16	53.8	61.16	44	39
2fg0	Glutamyl-diamino Endopeptidase	$P4_{1}2_{1}2$	1.79	0.154	3977	428	68,770	63.86	57.0	63.86	37	35
2uxj	Photosynthetic Reaction Center	$P4_{3}2_{1}2$	2.25	0.194	7707	403	98,164	76.0	72.1	76.56	34	28
3rd5	Mycobacterium Paratuberculosis	$P2_{1}2_{1}2_{1}$	1.5	0.133	2549	413	69,906	59.52	51.9	65.0	44	44
3tqe	Malonyl-CoA ACP Transacylase	C121	1.5	0.146	2915	347	72,567	56.29	48.0	63.07	45	41
4bex	Human Cofilin	$P3_{2}21$	2.8	0.207	1310	16	9292	74.99	70.3	75.04	43	35
4bsj	VEGFR-3 Extracellular Domains	$P3_{1}21$	2.5	0.21	1722	21	17,525	76.2	71.7	74.1	46	38
4gtf	Thymidylate Synthase	$I4_{1}22$	1.77	0.162	2086	124	34,764	60.72	53.3	63.38	37	31
4tpl	West Nile Virus Non-structural Protein	P321	2.9	0.17	5767	62	33,918	73.74	68.8	72.5	42	33
6g6b	Six-helix Coiled-coil Peptide	$P4_{1}32$	2.3	0.217	690	14	9242	75.02	70.3	74.99	40	33
7ubt	STAT5a Core with Compound	H32	2.35	0.201	4944	439	49,238	71.37	66.0	72.98	35	31
8x1l	S96C/L132C Mutant of FfIBP	$P4_{1}22$	2.0	0.227	1655	103	30,097	72.99	67.9	70.23	43	36
8xc8	Beta-1,4-galacosyltransferase	$P4_{1}2_{1}2$	2.51	0.236	3848	6	12,767	70.87	65.4	67.98	-	-
8yvo	C. Difficile Toxin A CROPs Domain	I121	2.1	0.21	3428	350	47,501	72.56	67.4	70.23	42	34
9ee7	S. Cerevisiae Xrs2’s folded Domains	$P6_{1}22$	2.38	0.298	2182	7	25,536	76.98	72.6	72.21	53	46
9eqq	Uhgb MS Mannoside Synthase	$P6_{1}22$	2.55	0.18	2909	160	25,524	73.16	68.1	74.38	38	30

**Table A2:** Comparison of direct phasing success rates (%) using HIO, HDM, and DiffMap on 22 protein structures under three schemes: Conventional, Resolution-weighted, and GA-enhanced.

	HIO			HDM_f1			HDM_f2			HDM_f3			HDM_f4			HDM_f5			HDM_f6			DiffMap		
PDB ID	Conv	Reso	GA	Conv	Reso	GA	Conv	Reso	GA	Conv	Reso	GA	Conv	Reso	GA	Conv	Reso	GA	Conv	Reso	GA	Conv	Reso	GA
1a53	0	2	100	0	0	0	0	17	100	14	42	100	9	59	100	0	0	0	0	0	0	0	0	0
1af2	40	32	100	41	51	100	35	42	100	46	49	100	44	42	100	30	45	100	34	58	100	38	34	100
1ass	28	26	100	26	13	100	19	12	100	24	20	100	22	14	100	46	35	100	36	23	100	4	0	0
1uii	35	41	100	16	33	100	31	30	100	32	36	100	29	39	100	0	29	100	8	29	100	0	6	100
1urm	2	4	100	11	13	100	20	20	100	8	10	100	10	10	100	1	15	100	7	9	100	0	0	100
1vmg	9	21	100	19	22	100	25	27	100	26	20	100	20	26	100	15	20	100	16	15	100	15	15	0
2evr	1	13	100	5	14	100	6	9	100	1	11	100	9	11	100	0	5	100	1	9	100	0	0	0
2fg0	0	1	0	0	0	100	1	0	0	0	2	0	2	1	0	0	0	0	0	1	0	0	0	0
2uxj	32	37	100	41	37	100	33	20	100	37	54	0	34	37	100	30	45	100	37	40	0	0	0	0
3rd5	1	1	0	0	0	0	1	0	0	0	2	0	1	0	0	0	0	0	1	0	0	0	0	0
3tqe	1	4	100	3	16	100	17	23	100	7	16	100	21	14	100	0	0	100	0	9	100	0	0	0
4bex	23	34	100	38	48	100	30	36	100	40	44	100	44	50	100	23	40	100	20	45	100	10	18	96
4bsj	2	10	100	0	2	100	0	1	100	1	1	100	0	0	100	0	1	100	0	3	100	0	0	100
4gtf	6	11	100	8	15	0	16	29	0	15	10	0	13	10	100	0	9	100	2	13	0	0	1	100
4tpl	0	1	100	0	0	0	0	0	0	1	1	0	1	1	0	0	0	0	0	0	0	0	0	0
6g6b	1	8	100	0	0	0	0	0	100	9	18	100	20	15	100	0	0	0	0	0	0	0	0	0
7ubt	0	5	100	0	0	100	0	1	100	0	1	100	1	2	100	0	0	0	0	0	100	0	0	0
8x1l	33	25	100	38	45	100	37	47	100	40	33	100	30	37	100	13	32	100	25	24	100	12	25	100
8xc8	0	0	0	0	0	0	0	0	0	0	0	0	0	0	0	0	0	0	0	0	0	0	0	0
8yvo	0	4	100	0	0	0	0	0	100	0	13	100	0	23	100	0	0	0	0	0	0	0	0	0
9ee7	12	11	100	5	5	0	7	6	0	10	6	100	8	5	0	7	15	100	13	7	100	2	6	100
9eqq	14	15	100	18	25	100	15	28	100	13	24	100	19	27	100	11	23	100	28	23	100	15	32	100

**Table A3:** Comparison of minimum iterations for successful convergence in direct phasing using HIO, HDM, and DiffMap on 22 protein structures under three schemes: Conventional, Resolution-weighted, and GA-enhanced.

	HIO			HDM_f1			HDM_f2			HDM_f3			HDM_f4			HDM_f5			HDM_f6			DiffMap		
PDB ID	Conv	Reso	GA	Conv	Reso	GA	Conv	Reso	GA	Conv	Reso	GA	Conv	Reso	GA	Conv	Reso	GA	Conv	Reso	GA	Conv	Reso	GA
1a53	-	2834	1803	-	-	-	-	635	679	3392	702	481	1123	1040	1065	-	-	-	-	-	1450	-	-	-
1af2	141	122	142	68	116	119	122	88	115	110	102	100	127	107	100	137	112	158	145	103	172	232	160	195
1ass	944	643	2971	3465	7166	3394	5448	6201	5570	2248	4650	1362	2978	3635	4955	302	2138	1583	493	2730	2537	1593	-	-
1uii	170	175	134	264	133	115	210	90	131	115	76	115	236	107	166	-	273	111	2394	180	181	-	431	415
1urm	5368	1370	802	1260	1698	1125	1204	894	946	683	1517	683	2212	1206	1026	6256	1289	1067	1587	2765	1133	-	-	2817
1vmg	489	363	374	312	282	174	267	431	363	244	253	254	257	299	174	431	350	231	619	565	340	1277	766	-
2evr	9042	1075	798	286	974	1199	770	852	595	6542	625	632	1386	955	640	-	2479	1098	7035	857	1129	-	-	-
2fg0	-	8007	-	-	-	4487	6521	-	-	-	2917	-	5040	7639	-	-	-	-	-	3415	-	-	-	-
2uxj	1845	1899	1705	1879	3065	1522	1500	4080	4957	1036	2176	-	1473	3105	1920	1716	2619	2992	1929	2575	-	-	-	-
3rd5	3926	4928	-	-	-	-	8148	-	-	-	976	-	3599	-	-	-	-	-	8312	-	-	-	-	-
3tqe	3783	5393	1610	3771	2013	1619	1413	2936	2305	1935	2469	1749	2507	2592	1287	-	-	1510	-	1614	1856	-	-	-
4bex	59	148	177	113	162	132	144	208	145	92	107	129	82	108	130	91	155	207	153	235	170	136	330	233
4bsj	2929	560	1186	-	590	350	-	275	300	331	251	562	-	-	961	-	4646	734	-	2487	486	-	-	1280
4gtf	2797	1926	1181	1160	1121	-	823	762	-	834	984	-	2231	1721	941	-	995	1629	1788	1994	-	-	8898	3014
4tpl	-	5475	1392	-	-	-	-	-	-	9086	7012	-	5563	9269	-	-	-	-	-	-	-	-	-	-
6g6b	8451	220	74	-	-	-	-	-	110	248	117	152	157	61	121	-	-	-	-	-	-	-	-	-
7ubt	-	1263	2069	-	-	1120	-	4980	1384	-	1683	2754	9219	7304	4253	-	-	-	-	-	2107	-	-	-
8x1l	288	271	187	177	396	291	193	476	273	229	221	271	273	258	452	738	381	227	277	259	486	1673	840	967
8xc8	-	-	-	-	-	-	-	-	-	-	-	-	-	-	-	-	-	-	-	-	-	-	-	-
8yvo	-	1070	935	-	-	-	-	-	484	-	544	381	-	732	758	-	-	-	-	-	-	-	-	-
9ee7	985	779	762	1326	170	-	650	478	-	1795	486	1186	861	472	-	1679	986	692	877	916	389	1681	924	1147
9eqq	241	533	398	154	658	428	208	553	758	479	523	385	162	524	474	338	428	442	277	411	475	915	713	388

**Table A4:** Comparison of median iterations for successful convergence in direct phasing using HIO, HDM, and DiffMap on 22 protein structures under three schemes: Conventional, Resolution-weighted, and GA-enhanced.

	HIO			HDM_f1			HDM_f2			HDM_f3			HDM_f4			HDM_f5			HDM_f6			DiffMap		
PDB ID	Conv	Reso	GA	Conv	Reso	GA	Conv	Reso	GA	Conv	Reso	GA	Conv	Reso	GA	Conv	Reso	GA	Conv	Reso	GA	Conv	Reso	GA
1a53	-	3493	1238	-	-	-	-	3995	814	6179	4258	1101	6271	3740	810	-	-	-	-	-	-	-	-	-
1af2	938	438	305	561	848	314	2113	520	402	3032	409	307	3838	426	302	754	716	312	519	363	353	1615	522	396
1ass	3044	7072	3402	6819	9033	4002	7863	8617	5906	7902	8551	2469	7248	8839	5401	4244	6788	2301	4760	8056	2902	7539	-	-
1uii	2505	671	320	6584	396	328	3421	458	316	3472	410	315	1967	398	316	-	1320	509	8236	590	411	-	1124	464
1urm	6930	1864	1102	4179	4070	1913	4284	3950	1427	4604	4917	1001	5750	4172	1037	6401	2894	917	6142	3913	1408	-	-	3135
1vmg	1134	1906	644	881	1185	552	786	948	704	1353	940	1443	1652	769	515	924	1782	506	1659	1956	626	2748	3363	-
2evr	9084	4767	1114	3313	3795	1602	4365	5416	1918	6568	6004	1162	3010	3596	1014	-	6184	1410	7104	5736	1303	-	-	-
2fg0	-	8070	-	-	-	4487	6562	-	-	-	5482	-	5334	7674	-	-	-	-	-	3467	-	-	-	-
2uxj	5306	4058	1904	5168	7022	1898	4682	8145	2682	3144	5392	-	4356	6686	1902	4449	5866	2515	6066	6788	-	-	-	-
3rd5	4099	5007	-	-	-	-	8178	-	-	-	4334	-	3653	-	-	-	-	-	8374	-	-	-	-	-
3tqe	3841	7270	1908	5222	7105	1612	4814	7161	2669	6922	6486	2120	5188	6483	1701	-	-	1901	-	4503	2201	-	-	-
4bex	722	4616	504	669	4701	316	1712	4657	406	1128	7352	306	932	5709	402	216	2843	501	880	2989	408	917	2674	722
4bsj	6148	2205	1407	-	640	786	-	336	703	347	284	922	-	-	1316	-	4661	1014	-	2967	803	-	-	1519
4gtf	4712	5013	1410	6190	2712	-	3287	3780	-	3720	2602	-	5805	3499	1408	-	3402	1901	4682	4047	-	-	8980	3503
4tpl	-	5569	1702	-	-	-	-	-	-	9503	7305	-	5698	9532	-	-	-	-	-	-	-	-	-	-
6g6b	8491	3320	503	-	-	-	-	-	328	1631	5044	504	1695	3686	401	-	-	-	-	-	-	-	-	-
7ubt	-	3401	2403	-	-	1504	-	5008	1803	-	1702	3104	9253	8224	4506	-	-	-	-	-	2402	-	-	-
8x1l	2032	5192	506	1311	9204	614	1227	9131	716	1038	9430	507	898	9416	805	2890	1737	508	1422	2392	802	5198	3756	1206
8xc8	-	-	-	-	-	-	-	-	-	-	-	-	-	-	-	-	-	-	-	-	-	-	-	-
8yvo	-	2406	1204	-	-	-	-	-	702	-	2174	708	-	3543	1001	-	-	-	-	-	-	-	-	-
9ee7	3014	2727	1118	4762	9154	-	2484	4762	-	4124	8983	1618	3172	4635	-	5363	4316	1014	3750	5050	870	5231	4456	1506
9eqq	592	1247	701	900	1917	836	823	2258	1001	856	1644	602	1156	2323	801	1386	1272	674	664	1742	701	2868	2651	678

## References

[1] Abramson J., Adler J., Dunger J., Evans R., Green T., Pritzel A., Ronneberger O., Willmore L., Ballard A.J., Bambrick J. (2024). Accurate structure prediction of biomolecular interactions with AlphaFold 3. Nature.

[2] Terwilliger T.C., Afonine P.V., Liebschner D., Croll T.I., McCoy A.J., Oeffner R.D., Williams C.J., Poon B.K., Richardson J.S., Read R.J. (2023). Accelerating crystal structure determination with iterative AlphaFold prediction. Acta Cryst. D.

[3] Li Z., Fan H., Ding W. (2024). Solving protein structures by combining structure prediction, molecular replacement and direct-methods-aided model completion. IUCrJ.

[4] Sayre D. (1952). The squaring method: A new method for phase determination. Acta Cryst..

[5] Cochran W.T. (1955). Relations between the phases of structure factors. Acta Cryst..

[6] Karle J., Hauptman H. (1956). A theory of phase determination for the four types of non-centrosymmetric space groups 1*P*222, 2*P*22, 3*P*_1_2, 3*P*_2_2. Acta Cryst..

[7] Schenk H. (1984). An Introduction to Direct Methods: The Most Important Phase Relationships and Their Application in Solving the Phase Problem.

[8] Miller R., DeTitta G.T., Jones R., Langs D.A., Weeks C.M., Hauptman H.A. (1993). On the application of the minimal principle to solve unknown structures. Science.

[9] Giacovazzo C., Siliqi D., Gonzalez Platas J., Hecht H.J., Zanotti G., York B. (1996). The *ab initio* crystal structure solution of proteins by direct methods. VI. Complete phasing up to derivative resolution. Acta Cryst. A.

[10] Sheldrick G.M. (2008). A short history of SHELX. Acta Cryst. A.

[11] Fienup J.R. (1982). Phase retrieval algorithms: A comparison. Appl. Opt..

[12] Elser V. (2003). Phase retrieval by iterated projections. J. Opt. Soc. Am. A.

[13] Elser V. (2003). Solution of the crystallographic phase problem by iterated projections. Acta Cryst. A.

[14] Millane R.P., Stroud W.J. (1997). Reconstructing symmetric images from their undersampled Fourier intensities. J. Opt. Soc. Am. A.

[15] Millane R.P. (1990). Phase retrieval in crystallography and optics. J. Opt. Soc. Am. A.

[16] Plas J.L., Millane R.P. (2000). *Ab initio* phasing in protein crystallography. Image Reconstruction from Incomplete Data.

[17] Miao J., Sayer D., Chapman H.N. (1998). Phase retrieval from the magnitude of the Fourier transforms of non-periodic objects. J. Opt. Soc. Am..

[18] Lunin V.Y., Lunina N.L., Petrova T.E., Urzhumtsev A.G., Podjarny A.D. (1998). On the *ab initio* solution of the phase problem for macromolecules at very low resolution. II. generalized likelihood based approach to cluster discrimination. Acta Cryst. D.

[19] Marchesini S. (2007). Invited Article: A unified evaluation of iterative projection algorithms for phase retrieval. Rev. Sci. Instrum..

[20] Su W.-P. (2008). Retrieving low- and medium-resolution structural features of macromolecules directly from the diffraction intensities—A real-space approach to the X-ray phase problem. Acta Cryst. A.

[21] Liu Z.C., Xu R., Dong Y.H. (2012). Phase retrieval in protein crystallography. Acta Cryst. A.

[22] He H., Su W.-P. (2015). Direct phasing of protein crystals with high solvent content. Acta Cryst. A.

[23] Millane R.P., Lo V.L. (2013). Iterative projection algorithms in protein crystallography. I. Theory. Acta Cryst. A.

[24] Lo V.L., Kingston R.L., Millane R.P. (2015). Iterative projection algorithms in protein crystallography. II. Application. Acta Cryst. A.

[25] Lo V.L., Kingston R.L., Millane R.P. (2016). Iterative projection algorithms for *ab initio* phasing in virus crystallography. J. Struct. Biol..

[26] He H., Jiang M.C., Su W.-P. (2019). Direct phasing of protein crystals with non-crystallographic symmetry. Crystals.

[27] Fu R., Su W.-P., He H. (2024). Refining protein envelopes with a transition region for enhanced direct phasing in protein crystallography. Crystals.

[28] Kingston R.L., Millane R.P. (2022). A general method for directly phasing diffraction data from high-solvent-content protein crystals. IUCrJ.

[29] Barnett M.J., Millane R.P., Kingston R.L. (2024). Analysis of crystallographic phase retrieval using iterative projection algorithms. Acta Cryst. D.

[30] Fu R., Su W.-P., He H. (2025). Genetic algorithm-enhanced direct method in protein crystallography. Molecules.

[31] Pan T., Dramko E., Miller M.D., Kyrillidisa A., George N.P. (2025). Completion of partial structures using Patterson maps with the CrysFormer machine-learning model. Acta Cryst. D.

[32] Millane R.P., Arnal R.D. (2015). Uniqueness of the macromolecular crystallographic phase problem. Acta Cryst. A.

[33] He H., Su W.-P. (2018). Improving the convergence rate of a hybrid input-output phasing algorithm by varying the reflection data weight. Acta Cryst. A.

[34] Winn M.D., Ballard C.C., Cowtan K.D., Dodson E.J., Emsley P., Evans P.R., Keegan R.M., Krissinel E.B., Leslie A.G., McCoy A. (2011). Overview of the CCP4 suite and current developments. Acta Cryst. D.

[35] Adams P.D., Afonine P.V., Bunkóczi G., Chen V.B., Davis I.W., Echoo ls N., Headd J.J., Hung L.-W., Kapral G.J., Grosse-Kunstleve R.W. (2010). *PHENIX*: A comprehensive Python-based system for macromolecular structure solution. Acta Cryst. D.

[36] Zhang K.Y.J., Main P. (1990). Histogram matching as a new density modification technique for phase refinement and extension of protein molecules. Acta Cryst. A.

[37] Afonine P.V., Grosse-Kunstleve R.W., Echols N., Headd J.J., Moriarty N.W., Mustyakimov M., Terwilliger T.C., Urzhumtsev A., Zwart P.H., Adams P.D. (2012). Towards automated crystallographic structure refinement with *phenix.refine*. Acta Cryst. D.

[38] Cowtan K. (2003). The Clipper C++ libraries for X-ray crystallography. IUCr Comput. Comm. Newsl..

[39] Wang B.C. (1985). Resolution of phase ambiguity in macromolecular crystallography. Methods Enzymol..

[40] Chojnowski G., Pereira J., Lamzin V.S. (2019). Sequence assignment for low-resolution modeling of protein crystal structures. Acta Cryst. D.

[41] Kovalevskiy O., Nicholls R.A., Long F., Murshudov G.N. (2018). Overview of refinement procedures within REFMAC5: Utilizing data from different sources. Acta Cryst. D.

[42] Cowtan K. (2008). Fitting molecular fragments into electron density. Acta Cryst. D.

[43] Terwilliger T.C., Grosse-Kunstleve R.W., Afonine P.V., Moriarty N.W., Zwart P.H., Hung L.-W., Read R.J., Adams P.D. (2008). Iterative model building, structure refinement and density modification with the PHENIX AutoBuild wizard. Acta Cryst. D.

[44] Koepke J., Krammer E.M., Klingen A.R., Sebban P., Ullmann G.M., Fritzsch G. (2007). Ph modulates the quinone position in the photosynthetic reaction center from *rhodobacter sphaeroides* in the neutral and charge separated states. J. Mol. Biol..

[45] Schrödinger, LLC (2025). The PyMOL Molecular Graphics System.

[46] Klumpp M., Baumeister W., Essen L.O. (1997). Structure of the substrate binding domain of the thermosome, an archaeal group II chaperonin. Cell.

[47] Nam Y., Nguyen D.L., Hoang T., Kim B., Lee J.H., Do H. (2024). Engineered ice-binding protein (FfIBP) shows increased stability and resistance to thermal and chemical denaturation compared to the wild type. Sci. Rep..

[48] Cioci G., Ladeveze S. Structure-Function Studies of Novel Bacterial Mannoside Synthase. https://www.rcsb.org/structure/9EQQ.

[49] Klejnot M., Gabrielsen M., Cameron J., Mleczak A., Talapatra S.K., Kozielski F., Pannifer A., Olson M.F. (2013). Analysis of the human cofilin1 structure reveals conformational changes required for actin-binding. Acta Cryst. D.

[50] Xiang S., Short S.A., Wolfenden R., Carter C.W. (1997). The structure of the cytidine deaminase-product complex provides evidence for efficient proton transfer and ground-state destabilization. Biochemistry.

[51] Vigneswaran A., Shi K., Aihara H., Evans R.L., Latham M.P. (2025). Crystal structure of the folded domains of Xrs2 from Saccharomyces cerevisiae. Acta Cryst. F.

[52] Saxena S., Yuan P., Dhar S.K., Senga T., Takeda D., Robinson H., Kornbluth S., Swaminathan K., Dutta A. (2004). A dimerized coiled-coil domain and an adjoining part of geminin interact with two sites on Cdt1 for replication inhibition. Mol. Cell.

[53] Baugh L., Phan I., Begley D.W., Clifton M.C., Armour B., Dranow D.M., Taylor B.M., Muruthi M.M., Abendroth J., Fairman J.W. (2015). Increasing the structural coverage of tuberculosis drug targets. Tuberculosis.

[54] Xu Q., Sudek S., McMullan D., Miller M.D., Geierstanger B., Jones D.H., Krishna S.S., Spraggon G., Bursalay B., Abdubek P. (2009). Structural basis of murein peptide specificity of a gamma-D-Glutamyl-L-Diamino acid endopeptidase. Structure.

[55] Elser V., Millane R.P. (2008). Reconstruction of an object from its symmetry—Averaged diffraction pattern. Acta Cryst. A.

[56] Kaneshige A., Bai L., Wang M., McEachern D., Meagher J.L., Xu R., Kirchhoff P.D., Wen B., Sun D., Stuckey J.A. (2023). Discovery of a potent and selective STAT5 PROTAC degrader with strong antitumor activity in vivo in acute myeloid leukemia. J. Med. Chem..

[57] Sluchanko N.N., Sokolova I.V., Favorskaya I.A., Esmagambetov I.B., Tukhvatulin A.I., Alekseeva I.A., Ungur A.S., Varfolomeeva L.A., Boyko K.M., Logunov D.Y. (2024). Structural insight into recognition of *Clostridioides difficile* toxin A by novel neutralizing nanobodies targeting QTIN-like motifs within its receptor-binding domain. Int. J. Biol. Macromol..

[58] Luo G., Huang Z., Zhu Y., Chen J., Hou X., Ni D., Xu W., Zhang W., Rao Y., Mu W. (2024). Crystal structure and structure-guided tunnel engineering in a bacterial beta-1,4-galactosyltransferase. Int. J. Biol. Macromol..

[59] Akey D.L., Brown W.C., Konwerski J.R., Ogata C.M., Smith J.L. (2014). Use of massively multiple merged data for low-resolution S-SAD phasing and refinement of flavivirus NS1. Acta Cryst. D.

[60] Leppanen V.-M., Tvorogov D., Kisko K., Prota A.E., Jeltsch M., Anisimov A., Markovic-Mueller S., Stuttfeld E., Goldie K.N., Ballmer-Hofer K. (2013). Structural and Mechanistic Insights Into Vegfr-3 Ligand Binding and Activation. Proc. Natl. Acad. Sci. USA.

[61] Direct-Phasing-of-Protein-Crystals-with-Hybrid-Difference-Map-Algorithms. https://github.com/hhe2/direct-phasing-of-protein-crystals-with-hybrid-difference-map-algorithms.

